# Metabolic interactions shape emergent biofilm structures in a conceptual model of gut mucosal bacterial communities

**DOI:** 10.1038/s41522-024-00572-y

**Published:** 2024-10-02

**Authors:** Amin Valiei, Andrew Dickson, Javad Aminian-Dehkordi, Mohammad R. K. Mofrad

**Affiliations:** 1 Molecular Cell Biomechanics Laboratory, Departments of Bioengineering and Mechanical Engineering, University of California, Berkeley, CA 94720 USA; 2 Molecular Biophysics and Integrative Bioimaging Division, Lawrence Berkeley National Laboratory, Berkeley, CA 94720 USA

**Keywords:** Microbiome, Biofilms

## Abstract

The gut microbiome plays a major role in human health; however, little is known about the structural arrangement of microbes and factors governing their distribution. In this work, we present an in silico agent-based model (ABM) to conceptually simulate the dynamics of gut mucosal bacterial communities. We explored how various types of metabolic interactions, including competition, neutralism, commensalism, and mutualism, affect community structure, through nutrient consumption and metabolite exchange. Results showed that, across scenarios with different initial species abundances, cross-feeding promotes species coexistence. Morphologically, competition and neutralism resulted in segregation, while mutualism and commensalism fostered high intermixing. In addition, cooperative relations resulted in community properties with little sensitivity to the selective uptake of metabolites produced by the host. Moreover, metabolic interactions strongly influenced colonization success following the invasion of newcomer species. These results provide important insights into the utility of ABM in deciphering complex microbiome patterns.

## Introduction

The gut microbiome, a rich and diverse microbial community that populates the human intestine, is considered an essential component of human physiology^1^. One of the most critical functions of the gut microbiome is the digestion of organic compounds, particularly those that cannot be metabolized by humans^2^. Thanks to their unique enzymatic capability, gut microbes can digest a variety of nutrients, including complex carbohydrates such as fiber, resistant starch, non-digestible polysaccharides, proteins, and bile acids^3,4^. The digestive action of the microbiome maximizes energy absorption during metabolism, and, in addition, metabolites derived from microbes promote human homeostasis^4,5^. Beyond the metabolic role, the gut microbiome is paramount to human immunity^1^. It is hypothesized that the bacterial colonization of the intestine provides protection against invading pathogens, a mechanism known as colonization resistance^6^. Furthermore, constant communication between the microbiome and host immune cells from early childhood is essential to training and modulating the immune system^7^.

The diversity of microbial roles within the gut has sparked key questions about the mechanisms of microbe-mediated functions. It is hypothesized that one of the major strategies by which gut microbes can perform complex roles is through the formation of bacterial communities^8^. While gut microbial consortia are known to interact in the floating mode in suspension, experimental data suggest that a noticeable portion of bacteria lives in close-packed communities called biofilms^8,9^. Biofilms are surface-attached aggregates of bacterial communities that form on surfaces and interfaces^10^. Attached bacteria in the biofilm mode undergo phenotypic and genotypic changes, applying unique abilities^10,11^. The growth of the cells within the biofilm matrix and the incorporation of additional cells result in unique three-dimensional structures^10,12^. Thanks to the physical proximity of the bacteria within a biofilm matrix, they can communicate intimately through the exchange of metabolites and chemicals. Furthermore, bacteria in biofilms attain remarkable resilience through the shielding effect offered by the biofilm structure^10,11^.

**Fig. 1:**
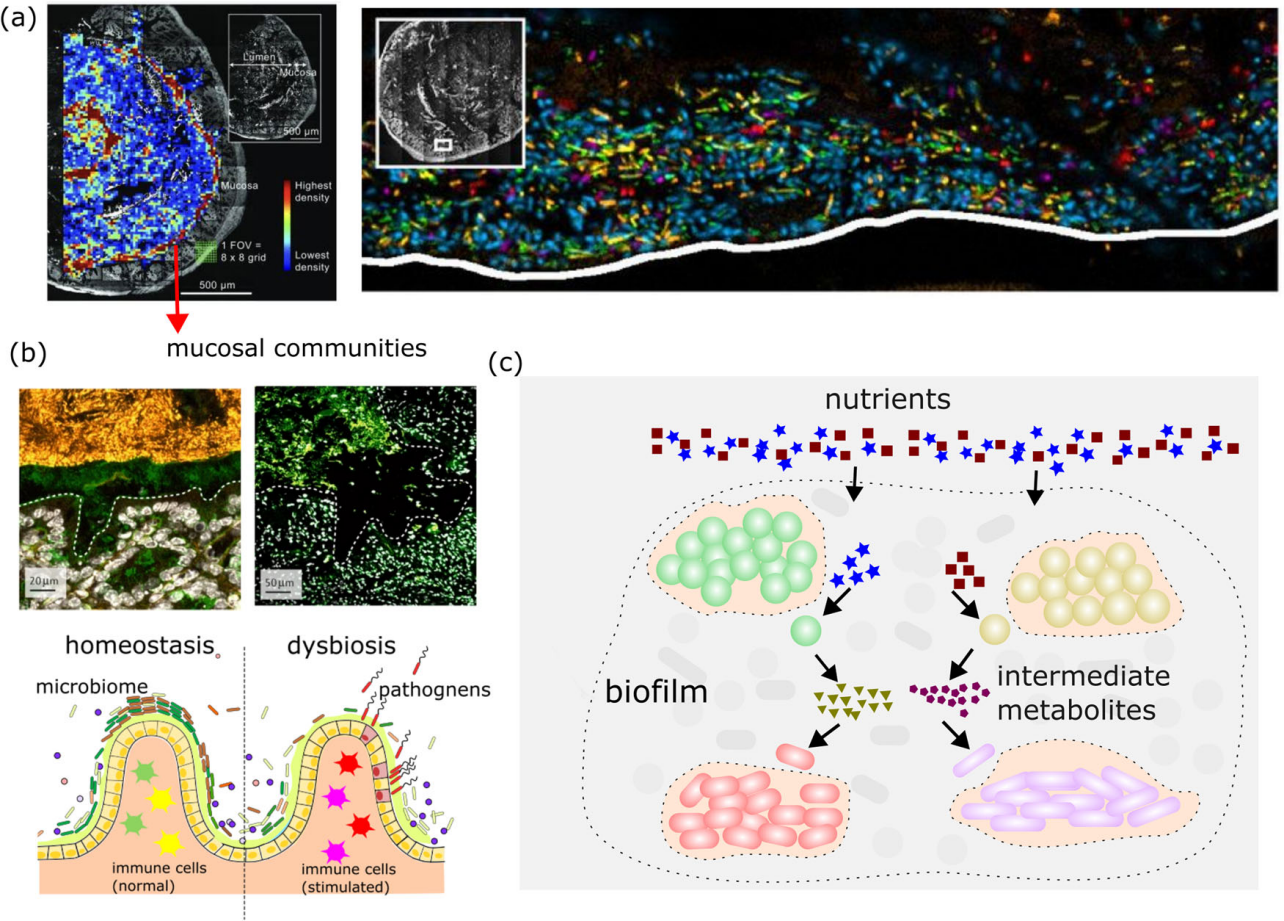
Gut mucosal microbiome. **a** (Left) The mouse colon consists of dense mucosal communities, as shown in the heat maps of bacterial density taken from lumen cross-sections. (Right) Mucosal communities in the mouse colon are compositionally diverse. Each dye refers to a different taxon. Adapted with permission from ref. ^42^. Copyright (2017) National Academy of Sciences. **b** (Top-left) In a healthy mouse gut, biofilm retains an integrated continuous structure over gut topographies; (top-right) in the diseased state, biofilm consists of dissociated aggregates. Gray stains host nuclei, green dyes glycoproteins, and yellow shows bacteria stained by 16S ribosomal DNA fluorescence in situ hybridization. Adapted with permission from ref. ^8^. Copyright (2018) Springer Nature. (bottom-left) The formation of a biofilm helps to maintain a homeostasis state; (bottom-right) the depletion of the biofilm layer makes the intestinal tissue exposed to pathogenic attacks (red bacteria), resulting in dysbiosis; this further causes inflammation by stimulating immune cells. **c** Schematic of microbial metabolic interactions in a biofilm. Biofilm bacteria interact through the uptake of nutrients and exchange of metabolic by-products.

Biofilm aggregates have been observed in various regions of the intestine, attaching to food particles in the lumen or on the surface of the gut mucosa, the latter in the form of dense layers known as mucosal communities (Fig. 1a)^8,9^. Recently, the functional characteristics of gut biofilm communities have gained significant attention from the scientific community, as they have been hypothesized to be important in metabolism, pathogenesis, and immunity^8,9^. Driven by multispecies interactions and patterns such as cooperation and competition, metabolic functionalities of biofilms diversify the digestion capability of nutrients^13,14^. The mucosal community is highly stable and long-lasting, which assures the microbiomes’s survival after the colon is subjected to antimicrobial treatments^8^. In terms of disease pathogenesis and immunity, it is seen that a healthy mucosal biofilm, featuring well-integrated structures, acts as a protective barrier against pathogenic attacks and contributes to an anti-inflammatory state^8^. In contrast, gastrointestinal diseases are often associated with compromised microbial structures, leading to infection susceptibility and chronic inflammation (Fig. 1b)^15,16^.

Despite the importance of gut mucosal biofilms, there remains a significant lack of knowledge regarding the structure and function of these communities, largely due to the limitations of existing research approaches. Sequencing techniques cannot profile bacterial spatial distribution and biofilm structures^17,18^; in vivo investigations are hindered by the difficulty of imaging live organisms^19,20^; and in vitro techniques are still nascent^21,22^. In this light, mathematical simulation and modeling offer a valuable avenue for gaining new insights into microbial communities^23^. One of the promising approaches for modeling microbial communities has been agent-based modeling (ABM)^24^, an effective paradigm for modeling complex biological systems^25^. Unlike traditional approaches that concentrate solely on population-level features, ABM adopts a holistic, bottom-up viewpoint, driving emergent system functionalities from the characteristics of agents^26^.

ABM has been previously used to model biofilms and bacterial communities in environmental and industrial problems^27,28^. Various biofilm ABM models have been built on different spatial-temporal details ranging from temporal microbial populations to community structures^27,29,30^. Specifically, ABM models, such as those developed by Momeni et al.^31,32^, Kang et al.^33^, and Schluter et al.^34,35^, are apt to elucidate structural patterns resulting from intercellular mechanisms such as cross-feeding and competitive interactions. Previously, ABM has been used to study the gut microbiome^36,37^, but most past works focused on populational aspects; few works have so far modeled spatiotemporal biofilm structures^34,38^. In this study, we combined ABM with the finite volume method (FVM) to model an intestinal mucosal biofilm niche with conceptual metabolic-mediated interactions (Fig. 1c). While this model is relatively sophisticated in terms of investigating the generic biofilm structure from individual agent interactions, it is not intended to be the full description of the gut microbial communities, as there is significant complexity in capturing biological and physicochemical processes within the system. Nevertheless, with this preliminary model, we show the importance of interspecies interactions, including competition, neutralism, commensalism, and mutualism, in shaping the biofilm structure and determining resistance against newcomer invasions. The findings set the stage for future experimental and mathematical modeling aimed at deciphering the characteristics of gut microbial communities.

## Results

### Metabolic-mediated populational and structural patterns in gut biofilms

**Fig. 2:**
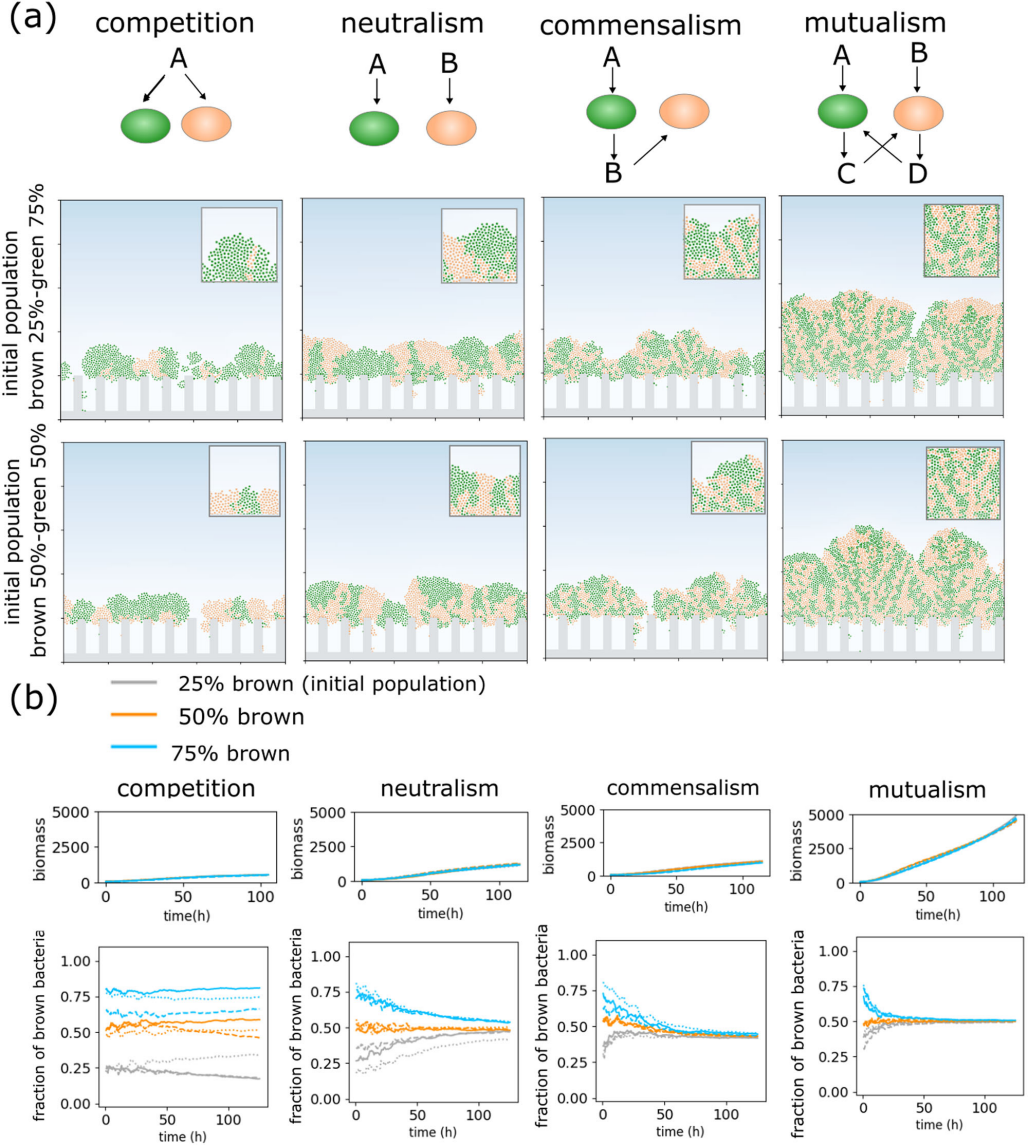
Metabolic interactions as a driver of mucosal biofilm structures. **a** Top schematics show the metabolic relationships between two bacterial species (green and brown). A, B, C, and D denote different nutrients; A and B are nutrients sourced from the bulk fluid, while C and D are generated during bacterial metabolism. The morphology of biofilms for each category of metabolic relationship after maturation (*t* = 120 h) is shown for different initial compositions; the inset shows a magnified view of cell positions. **b** Graphs showing the biomass growth and the relative abundance of brown bacteria over time. Each line type (solid, dashed, and dotted) represents a repetition, and each line color corresponds to an initial population composition.

Herein, we present a conceptual ABM model of biofilms that can derive the effect of localized microbial interaction over time and in space. In the model, each bacterium is represented as an agent that is able to attach to the surface, grow, replicate, and shove neighboring bacteria. Interactions among bacteria and between bacteria and the environment are mediated by the consumption of metabolites and nutrients (refer to “Methods”). By superimposing the physics underlying the chemical interactions, including diffusion and biochemical reactions (Monod kinetics^39^), we employ a framework to obtain emergent community properties from various interaction types. We investigate the following fundamental relationships in a bacterial community (Fig. 2a): (1) competition, where bacteria compete over a common nutrient; (2) neutralism, where each bacterium utilizes a different distinct nutrient, with no metabolic interaction from others; (3) commensalism, where one type of bacterium feeds on by-products produced by another; and (4) mutualism, where bacteria feeding on different nutrients also consume metabolic by-products produced by one another (also known as cross-feeding). These interactions are key to understanding the dynamics of the gut microbial community^40,41^. We first simulated these interactions in dual-species biofilms growing in a mucosal niche. We developed our model toward elucidating generic patterns of a model mucosal biofilm from each interaction type without aiming for rigorous species-specific models.

In our first set of simulations, bacteria were given symmetrical metabolic characteristics involving identical growth constants, both for nutrient uptake from the bulk fluid (lumen) and for the consumption of metabolic by-products. Bacteria were set to grow under diffusional constraints with a constant concentration of bulk nutrients at the top boundary. The simulations illustrated bacterial growth resulting in a mature, structured biofilm over protruding topographies, representing the crypts, within several days (Fig. 2a). Simulations were cut off at timesteps, producing biofilm thicknesses ranging from 50 μm to 200 μm, reflecting the range observed in in vivo gut biofilm images^8,42^ Bacterial growth occurred under a significant nutrient gradient, with the majority of growth occurring at the topmost layer of the biofilm and little growth within the crypt spacings.

Comparing biofilm structures across different scenarios revealed substantial differences in biofilm morphologies (Fig. 2a). While it was observed that biofilm structures feature growth both vertically and laterally, their architectural characteristics varied considerably. Biofilms developed under competition consisted of sparse, segregated patches, while those arising from neutralism were composed of separated but larger patches. In contrast, bacterial cross-feeding caused substantial intermixing, resulting in small, interconnected sectors. Interestingly, these distinct structural patterns emerged as signatures of each interaction, persisting even when initial abundances were altered (Fig. 2a). Temporally, these patterns appeared early in biofilm evolution and endured throughout its life cycle, indicating a rapid tendency to adopt distinct morphologies corresponding to each interaction type (Supplementary Fig. 1).

Metabolic interactions were further demonstrated to influence populational aspects (Fig. 2b). In the competition scenario, the final biofilm composition was similar to the initial relative abundances, whereas in other cases, relative abundances tended to converge to a common value. In these simulations, the amount of nutrient consumption, and, therefore, the biomass growth rate varied depending on the interaction type. For instance, mutualistic bacteria in our model benefit from a larger absorption of combined bulk nutrients and metabolic by-products. Thus, to ensure structural and populational features remain independent of the biomass content, we performed additional case studies. Firstly, we compared biofilm structures at the same biomass content (but at different time points, including the hypothetically extended durations). This simulation showed that the population ratios and structural features specific to each type of interaction were preserved. Supplementary Fig. 2a, b, showing the morphology of biofilms at equivalent biomass levels, illustrates tall, and segregated structures in the competition scenario, as seen in other studies^28,43^, which dramatically differ from flat and interconnected domains in the cross-feeding cases. In the second approach, we enforced equal biomass (at a specific time point) across scenarios by scaling down the growth rate in faster-growing biofilms (Supplementary Fig. 3). This also showed that the general populational and morphological properties were conserved. Bacterial species were initialized in both random and alternating patterns, but neither distribution produced noticeably different biofilm patterns. (Supplementary Fig. 2c).

**Fig. 3:**
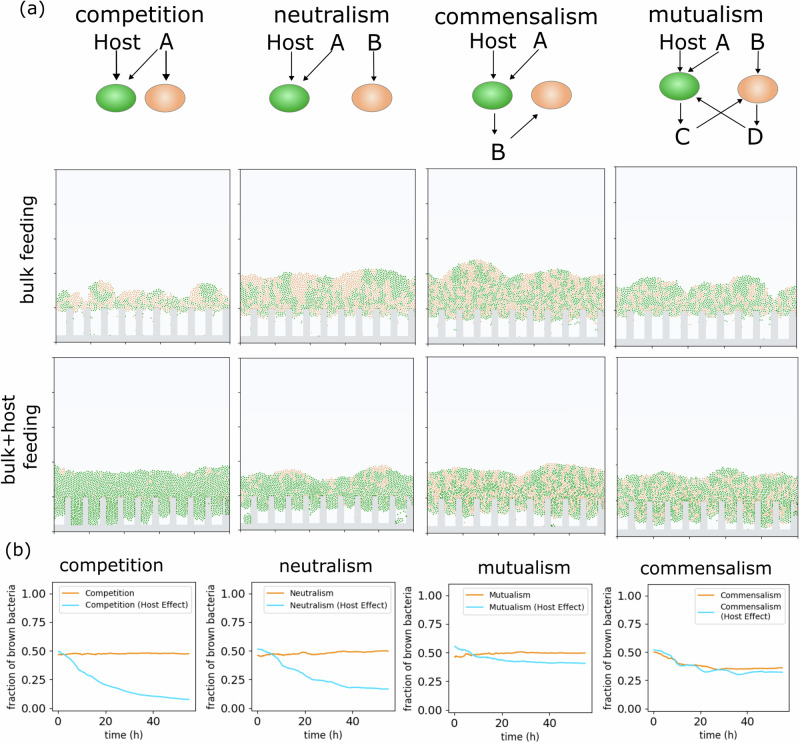
Effects of host secretions on bacterial communities in the cases of competitive, neutral, mutualistic, and commensal community interactions. For compatibility with existing simulations, we modeled the host effect as an additional metabolite, diffusing from a constant concentration source at the host surface. In an extreme scenario, we assumed gut wall solute concentrations are 20% of the maximum bulk nutrient concentration at the top system boundary (lumen), and that host secretions were consumed by green bacteria. **a** Morphology of biofilm structures for various metabolic relationships at the 50%–50% initial bacterial abundances. **b** Relative abundance of brown bacteria over time for each of the modeled interactions, with and without host effect. The simulation parameters for each bacterium were the same as the baseline simulation (Fig. 2).

Since the goal of this conceptual biofilm model was to understand various aspects of gut mucosal communities, we next evaluated the effect of the host, which, situated under the mucosal biofilm, is considered an important factor in gut biofilm microbiology. The host is known to establish symbiotic relationships with microbes in a selective manner. Host selection occurs through various means, notably, chemical interactions mediated by the metabolites it produces. The differential ability to metabolize host secretions can modify the composition of bacteria adjacent to the epithelium, which may influence the overall biofilm structure. To assess this mechanism, we explored biofilm growth in the presence of metabolites produced by the host in addition to those diffused from the bulk fluid at the top boundary (Fig. 3). Observing morphological similarity to the preceding results for each interaction (Fig. 3a), we see that bacterial abundance resulting from competition is strongly influenced by the bacterial bottom layer affected by the host, while the structure of cross-feeding populations remains relatively independent of this effect (Fig. 3b). This trend holds even when the concentration of host metabolites is relatively large (20% of the nutrient concentration at the top boundary), driving a strong preference for one strain. These results are interesting as, although previous models also emphasized the major role of the host in competition^34,44^, we demonstrate that such a role is alleviated in cross-feeding communities. Overall, both qualitatively and quantitatively, bacterial metabolic interactions emerge as a key player in shaping gut biofilm communities.

Considering the importance of these results, we searched for alternative factors in our model that may impact the biofilm structure. A candidate factor was the surface topography, which is composed of crypt structures of the intestinal wall^45^. Experimental data show that planktonic bacteria interact with microstructures in various ways^46–48^. Microscale cavities can provide selective adhesion sites for more motile bacteria^49,50^ and offer protection against predators or environmental stressors^48,51^. To model the effect of topography, we simulated the biofilm formation after increasing the motility or varying the cavity size; however, we observed little impact on biofilm dynamics. Although bacteria with higher motility tended to increase the surface attachment inside cavities, metabolic interactions rather than attachment patterns played the dominant role (Supplementary Fig. 4). Variation in the spacing between the micropillars, a key geometric feature, also had negligible influence on growth and abundance curves due to significant mass transfer limitations (Supplementary Fig. 5; also see the biofilm simulation on a flat control surface in Supplementary Fig. 6). In an in vivo setting, bacterial colonization within crypts is confounded by the repelling effect of the mucin layer secreted routinely by the epithelium (not simulated here). Certain bacterial species, however, have demonstrated the ability to degrade and penetrate through this layer within the crypts^42^. Our results show that, for these bacteria, mass transfer limitations pose a significant hindrance to growth and space occupation in this region.

### Variation of metabolic parameters

**Fig. 4:**
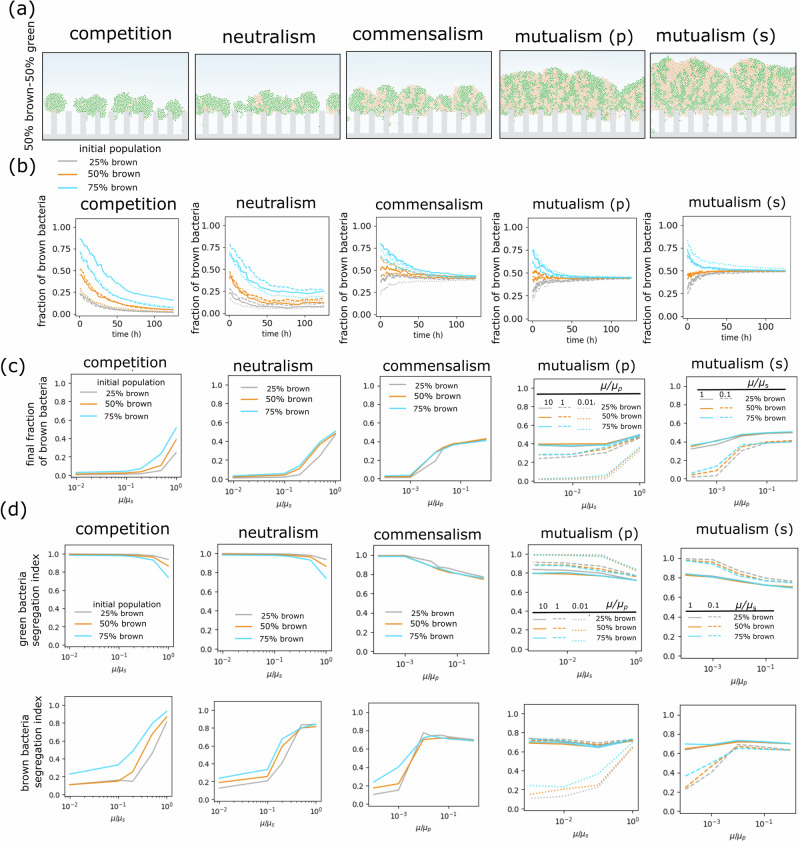
Effects of metabolic parameters on the growth and structure of simulated gut biofilms. **a** Morphology of biofilm structures for various metabolic relationships at the 50%–50% initial relative abundance. *µ*_max_ (brown) = 0.25 *µ*_max_ (green). The (p) and (s) notations refer to the cases with reaction rate variation for metabolic by-products and bulk nutrients, respectively. **b** The relative abundance of brown bacteria over time for various specific growth rates. **c** The relative abundance of brown bacteria was computed over multiple orders of magnitude of growth rates. (s) and (p) labels are the same as (**a**). **d** The segregation index for green and brown bacteria at different growth rate conditions.

Considering the important role of metabolic interactions in biofilm dynamics and properties, we conducted further in-depth case studies on the biofilm structure. We sought an assessment of biofilm growth following asymmetrical growth rates between two bacteria. Figure 4a, b shows morphological and populational differences caused by reducing one specific growth rate to 25% of the other. In competitive interactions, regardless of the initial abundance, the biofilm structure exhibited the disappearance of slower-growing bacteria along with a significant loss in biomass growth and integrity. Conversely, growth rate variations had a less notable impact in non-competitive regimes (neutralism, commensalism, and mutualism). For neutralism, despite a decline in the slower-growing strain, extinction remained improbable. For mutualism, regardless of the initial population, the solution tended to stabilize at an equal relative abundance.

Comparing relative abundances across multiple orders of magnitude of specific growth rates (Fig. 4c) shows that despite perturbations in primary nutrient or metabolite uptake rates, the final relative abundance in the mutualism remained relatively unaltered. On the contrary, competition exhibited the highest vulnerability, as even a marginal asymmetry resulted in the elimination of species with a smaller growth rate. While the difference in growth rate can be caused by inherent biological differences between species, it can also be impacted by external conditions such as oxygen concentration. The crypt axis particularly features an oxygen gradient, which can affect the growth rate of bacteria^52^. We, therefore, conducted a preliminary simulation on the impact of oxygen conditions on biofilms (refer to the Supplementary Information). The variation of growth rates with oxygen for the two types of bacteria were assumed to be either similar or widely different. In the former, changes in growth rate did not cause a noticeable impact on the biofilm pattern and composition (Supplementary Fig. 7). In the latter, competition resulted in the dominance of the bacteria with a faster specific growth rate, and mutualism featured the least variation in populational and structural patterns (Supplementary Fig. 7). The impact of metabolism on diversity and robustness, the ability to maintain structure in varying environments^53^, was also seen in the case of variations in nutrient concentrations (Supplementary Fig. 8). The presence of cross-feeding in mutualism ensured higher robustness compared to neutralism.

To better analyze the biofilm structures derived from these scenarios, we next assessed the interspersion of species, a vital factor in understanding their distribution within biofilms. Here, drawing on the Schelling social interaction model^54^, we defined an index that quantifies segregation by measuring the abundance of neighboring bacteria (*n* = 8) for each bacterium based on the Euclidean distances. The value of the index, averaged over the entire population for each species, ranges from 0 to 1. The index represents higher clustering for high values and greater intermixing for low values. As shown in Fig. 4d, the segregation index for various scenarios revealed a strong dependence on the community metabolism. For the baseline metabolic scenario, the segregation was highest for the competition case (0.8 for brown—0.9 for green) and depended on the initial relative abundance. In contrast, in non-competitive interactions, the segregation was lower and followed a nearly symmetrical distribution (neutralism: ~0.85, commensalism: ~0.70, and mutualism: ~0.70, values are for both bacterial types). Interestingly, an asymmetrical growth between the two bacterial types resulted in a disparity between indices, except in the case of mutualism, where a stable segregation index was sustained over several orders of magnitude changes in growth rate. This suggests that cooperative metabolic exchange supports structural stability—the ability to maintain a balanced state following a perturbation^55,56^. These findings reinforce that metabolic interactions within biofilms are determining factors in their architecture and their response to variations in kinetic features and environmental factors.

### Polymicrobial biofilms and invading bacteria

The dynamic interactions among bacteria typically involve intricate and complex metabolic networks among several species. From a bottom-up perspective, these networks encompass various pairwise interactions, such as competition, neutralism, commensalism, and mutualism. Supplementary Fig. 9a, b illustrates the expansion of previous interaction scenarios into three-species communities. We observed that the qualitative and quantitative features of two-way interactions, such as intermixing and relative abundance patterns, are largely preserved in more diverse populations, including the contrasting dynamics of competition and cooperation.

**Fig. 5:**
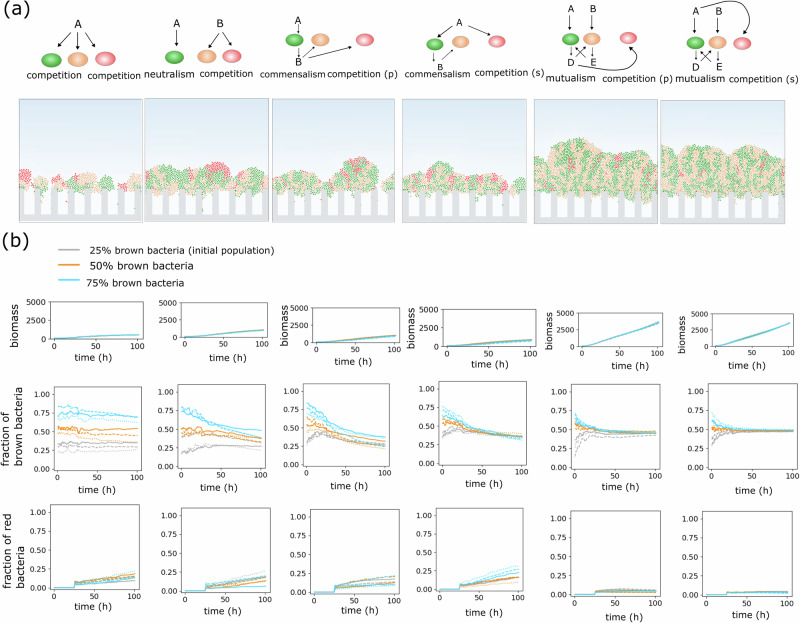
The gut biofilm simulations with three bacterial species. The third bacteria (red) are introduced at *t* = 25 h, during the two-species biofilm growth (brown and green). **a** Schematic of the metabolic relationships and the morphology of biofilms at *t* = 120 h. The notations (p) and (s) indicate the competition over metabolites produced by bacteria and bulk nutrients, respectively. The initial number of bacteria is 200, and the population of the invading bacteria is 100. The bacterial kinetics parameters are the same as the baseline simulation (Fig. 1, also refer to Supplementary Table 1). **b** Temporal changes of biomass and the relative abundance of brown and red bacteria within biofilms.

As the number of bacterial species increases, new scenarios emerge resulting from the combination of different pairwise interactions. An important potential scenario would be when an added species introduces a different interaction type to the ecology. Understanding whether the added bacteria can establish a foothold will shed light on the susceptibility of a community to imbalances, such as the introduction of pathogens, and its propensity to maintain homeostasis. To delve deeper into this problem, we conducted investigations involving the addition of a third species in two distinct studies. In the first one, a third species of bacteria invades a pre-existing biofilm layer, reflecting the dynamics of bacterial modifications when a mature microbiome encounters a new microbe. The second study focuses on the grassroots colonization of three species within a niche. These results are shown in Fig. 5 for a two-species microbiome inoculated at 25 h by a third species (red) and Supplementary Fig. 10 for the cocolonization of three bacteria. All bacteria have kinetic properties identical to the baseline scenario.

Figure 5 shows that when the invading bacteria are in a competitive relationship with an existing biofilm featuring competitive, neutralistic, or commensal communities, they proliferate considerably at the upper biofilm layer. Mutualism is the only scenario where the third species is inhibited. In the case of cocolonization, the third bacteria also grow substantially in all cases, with the least growth occurring in mutualism (Supplementary Fig. 10). When the invading species establish a cross-feeding (commensalism, mutualism) relationship with the existing bacteria, not only are they able to establish a foothold within the community, but they can also invade through the biofilm due to the significant intermixing effect (Supplementary Fig. 11). Unlike the competition case, the bacterial fractions attain a steady state that enables the coexistence of all the species.

These findings shed light on the significance of metabolic interactions in the dynamics of microbial communities, particularly in determining the outcome of pathogenic invasions or microbial interventions focusing on the microbiome modification. Currently, there is substantial ambiguity in the identification of successful invading factors^57,58^. Bacterial properties like chemotaxis, motility, host affinity, and metabolic factors have been hypothesized to be crucial to colonization success^57,59,60^. In the conventional notion of colonization resistance, nutrient limitation is considered an important factor against the establishment of newcomers^61,62^. In this context, our findings reinforce the significance of the spatiotemporal aspect of microbial metabolic interactions^62,63^. Benefiting from a much higher growth rate in the upper biofilm layers, newcomers with sufficiently strong competition can thrive and dominate the population. An alternative strategy to gain a deeper foothold in a biofilm, however, may involve utilizing metabolic by-products. This has profound implications for community succession, as cross-feeding newcomers introduced into the biofilm over time can continually enhance the cooperative networks inside the system.

**Fig. 6:**
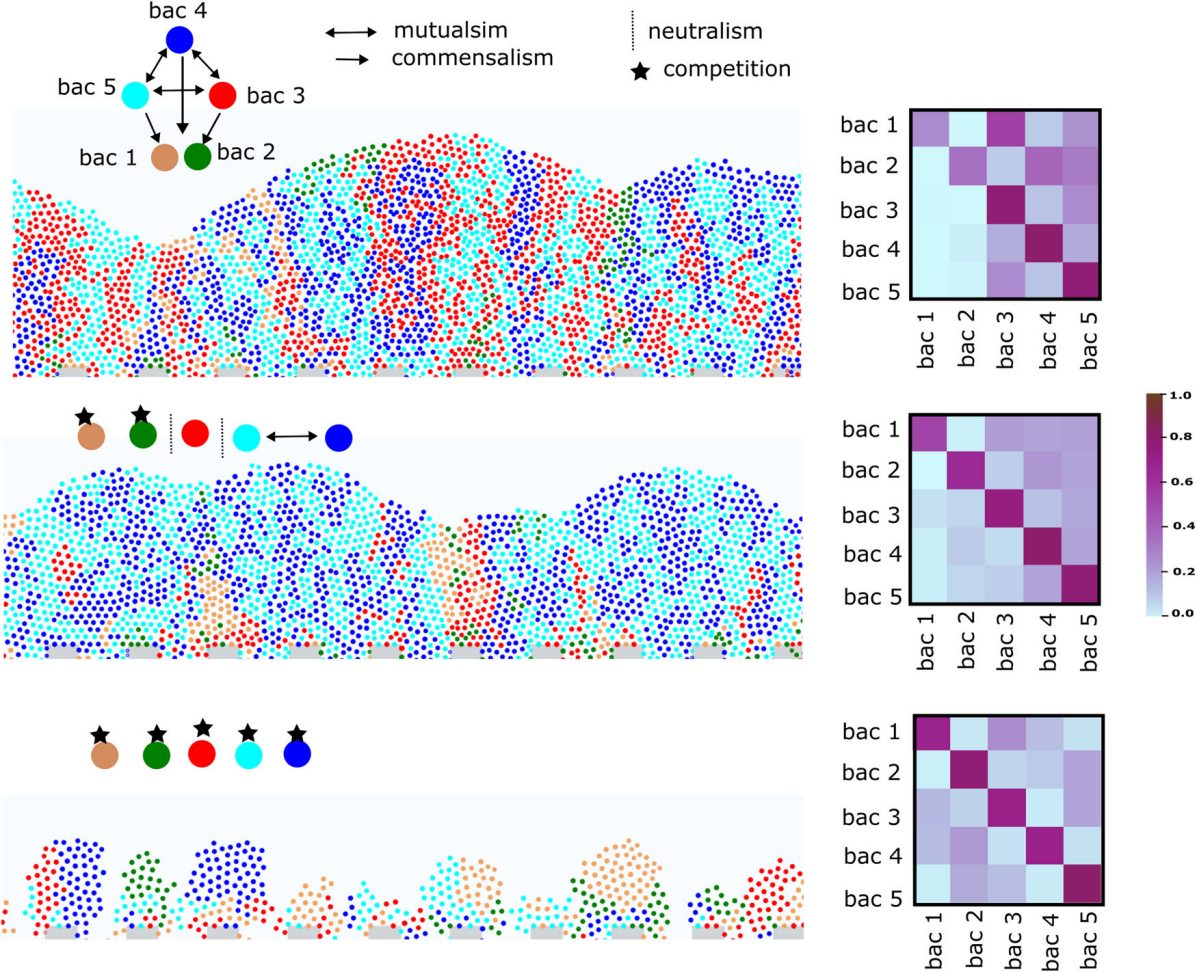
Spatial distribution of the gut microbial community resulting from ABM simulations of a multi-species biofilm on a modeled intestinal niche. (Upper) A highly cross-feeding community, including three species in bidirectional cross-feeding and two species in unidirectional (commensal) cross-feeding. Most bacterial species are inter-dispersed. Also, see Supplementary Fig. 12 for schematics showing detailed bacterial relationships and further details. (Middle) A community featuring one cross-feeding pair, one competitive pair, and a neutralistic species. The competitive bacteria, which grow more slowly, are outcompeted. (Lower) A purely competitive community. The dispersion, especially at the upper growth front, is relatively small. The right-hand side heatmaps show the segregation index matrix for each type of interaction (for the definition of segregation matrix refer to “Methods”).

Based on the insights obtained on the dynamics of multispecies communities so far, a relevant question is whether it is possible to quantitatively correlate the biofilm structure in the theoretical model with metabolic interaction networks in cases with larger numbers of species. Through additional simulations (Fig. 6) conducted on five species, we discovered that the spatiotemporal properties of microbes in biofilms with a larger number of species can result in distinct patterns, providing a means to analyze and infer interspecies interactions. We defined a segregation matrix for polymicrobial systems whereby each matrix element represents the pairwise segregation index between the corresponding species (refer to the “Methods”). We then calculated this matrix for various initial attachment patterns. Figure 6 and Supplementary Fig. 12 show that while, to some extent, dependence on different initial deposition patterns is observed, the segregation matrices computed for multiple repetitions remain similar. Overall, mutualistic bacteria tend to disperse better, while competitive systems have a strong preference for segregation (Fig. 6).

In essence, our model underscores the significance of metabolic interactions in biofilm structural patterning. However, it is crucial to recognize that alternative mechanisms, beyond the scope of our model (refer to “Discussion”), may also play a role in biofilm morphology. Consequently, conducting thorough experimental analyses and developing rigorous models is imperative to obtain conclusive insights into these systems.

### Comparative analysis of the results with population-based approaches

Investigating the ABM model in various scenarios shows that this method can uniquely predict biofilm’s morphological properties while also computing populational metrics. To elucidate the significance of emergent spatial heterogeneity within ABM, we compared the quantitative findings with a conventional ordinary differential equation (ODE)-based model of microbial populations. The ODE was constructed according to a transient reaction-diffusion equation based on the assumption of nutrient mass transfer across a similar diffusion length equal as ABM. This definition ignores the spatial effects across the biofilm, assuming a well-mixed system. Just like previous case studies, we analyzed the growth and stability of microbial communities in scenarios involving different initial abundances and specific growth rates as well as the addition of newcomers.

**Fig. 7:**
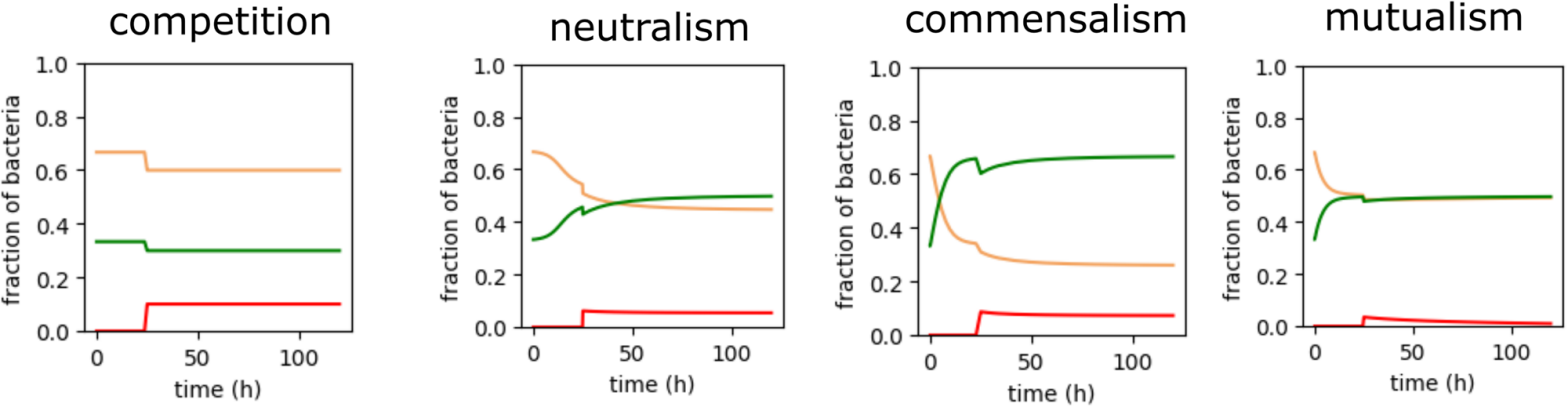
Results of a population-based model simulating various types of metabolic interactions. Effect of the invasion of newcomers (red) on populational abundance within the biofilm community (red and brown in competition).

As depicted in Supplementary Fig. 13, ODE results acquired from different initial abundances with bacteria having identical metabolic growth are in qualitative agreement with ABM, although they tend to overestimate the growth rates. The ODE model, in addition, exhibits limitations in simulating spatial or temporal events. We compared the competing-newcomer-invasion scenario at *t* = 25 h in the ODE simulation with the previous ABM results (Fig. 7 and Supplementary Fig. 14). Injecting the third type of bacteria in ratios similar to the previous scenario led to negligible changes in population or complete depletion in all scenarios. This difference primarily stems from the neglect of physicochemical gradients in ODEs, as ABM results show that the majority of growth occurs at the upper biofilm layer. While PDEs could alternatively be used to simulate the physiochemical gradients, they lack the means to derive bacterial patterns such as intermixing. It could thus be inferred that the proposed ABM modeling (including an FVM PDE solver) has substantial advantages in deriving spatiotemporal populational effects and is highly suitable for acquiring mechanistic insights involving microbial interactions into biofilm communities.

## Discussion

The experimental study of the gut microbiome has always been associated with challenges due to the inaccessibility of the digestive tract. Theoretical models, as an alternative approach, can provide insights into experimental observations, help form hypotheses, and make predictions in scenarios that are difficult to replicate in the laboratory^26^. A meritorious modeling approach suitable for understanding microbial communities is ABM, as it enables the driving properties of communities through spatiotemporal microbial interactions^24,27^. While the combination of ABM and a PDE solver (such as FVM) has been used previously to model bacterial behavior^29^, we pivoted this approach to study biofilm growth in a conceptual gut mucosal model. We modeled the metabolic interactions, representing competition, neutralism, and cooperation, and realized distinct growth patterns for each type of interaction. Morphologically, we observe patchy structures in non-cooperative interactions and well-intermixed configurations in cooperative ones. In the latter case, both structural and populational characteristics were highly preserved when the metabolic rates were perturbed or when the initial conditions were changed. These properties were found to be valid among the various scenarios we studied for each interaction type.

Previous works have also studied the interaction of microbes using agent-based approaches^31^^,^^64^. Blanchard et al. studied the radial expansion of binary colonies grown following a central deposition at various social interactions, including the ones studied here^64^. Similar to the conclusions drawn here, the researchers found that mutualism and commensalism reduce the variance in relative abundance and promote structural smoothness and homogeneity^64^. Our work also aligns with the pioneering work of Momeni et al., who observed that mutualistic relations promote microbial intermixing both experimentally and with an ABM model^31^. These models, despite having differences in problem formulation, geometry, and interaction models, conclusively highlight the importance of intercellular interactions in community structures. Building on these insights, our results suggest that ABM models can also be incredibly useful in understanding biofilm properties in the context of gut mucosal communities.

Our model particularly provides new ecological perspectives on gut bacterial communities relevant to the microbiology of humans and potentially other species. Among various issues, the relative dominance of cooperation vs. competition has been the subject of extensive discussions in multiple studies^65,66^. Although competition generally has been suggested as a better survival strategy^65,67^, collective behaviors such as biofilm formation, quorum sensing, and cross-feeding have been observed in bacterial communities^68,69^. Our simulations indicate the importance of additional factors, such as structural features when comparing different classes of interactions. Interspersion and structural homogeneity conferred in mutualistic communities can impart resilience and integrity. Additionally, mutualistic relations are extremely successful in dampening compositional variations and establishing footholds for mutualistic newcomers. This uncovers an important fitness advantage for cross-feeding networks in gut microbial systems.

These findings can be used to develop important hypotheses on various aspects of the gut microbiome. For instance, while it is generally known that the composition of the microbiome remains relatively stable in adults, the factors governing such stability are still unclear^70,71^. Temporary dietary changes or interventions like antibiotics and probiotics cause short-term variations, but the microbiome typically returns to its original state^70^. On the other hand, it is suggested that mucosal populations are important in the colonization and establishment of the microbiome and are hypothesized to contribute to its retention, among other potential mechansims^72–76^. In this respect, having a more diverse and well-distributed mucosal community, as seen in mutualistic interactions, can potentially contribute to a more functional and diverse microbiome composition.

While the ABM model has captured the importance of different modes of metabolic interaction, the gut ecosystem is generally too complex, and other mechanisms may impact bacterial interactions and biofilm structures. In this study, we did not account for fluidic effects considering the low flow rate in the large intestine (Reynolds number ~0.3)^75,77,78^, however, modeling convective mass transfer and shear forces can enhance the accuracy of biofilm models in the future, particularly when extending the simulations to the upper gastrointestinal tract. The incorporation of hydrodynamic effects can also help model bacterial dispersal behavior, as mucosal bacteria are affected by cycles of attachment and detachment which can influence the biofilm structure. Furthermore, stochastic events can also influence the overall structure of biofilms. For example, we have shown that the initial position of bacteria can impact the outcome when the number of species increases. Developing more rigorous models of biofilm structures and using statistical techniques to analyze them may provide a rigorous link between model outputs and experimental data. Moreover, the effect of mucus production, not modeled here, is suggested as an important factor in gut biofilm dynamics. Mucus can particularly hamper the biomass growth in crypts, as seen in microscopic images^42^. Lastly, a better understanding of biofilm physical mechanisms, such as those involving death and shrinkage, can help improve the model’s fidelity in the future. Obtaining experimental data on biofilm properties and metabolic parameters is further essential to improving the models. Together, the platform developed in this study represents an advantageous tool for gaining insights into the dynamics of microbial communities in the gut. None of the findings on biofilm morphology and spatiotemporal populational properties were possible to obtain from conventional modeling approaches such as ODE. The integration of computational and experimental approaches holds great promise for advancing our understanding of gut microbial dynamics and its broader applications.

In conclusion, in this work, we presented a conceptual ABM of competitive and cooperative microbial interactions within a biofilm system designed toward deciphering the gut mucosal microbial community. The model simulated these interactions through simplified biochemical reactions involved in the consumption of bulk nutrients and metabolic by-products. The four interactions studied included competition, neutralism, commensalism, and mutualism. We found that these interactions guide the growth and spatiotemporal variation of the community, resulting in structural and behavioral patterns that were valid across a wide range of growth ratios, nutrient concentrations, and initial abundances. We notably found that competitive and neutralistic relationships promote segregation and structural discontinuity, while commensalism and mutualism cause maximal biomass interspersion and growth. We further found that mutualistic relationships were more resistant once additional species were incorporated into the microbial mixture or introduced into mature biofilms. Taking advantage of the higher concentration of nutrients in void areas or the topmost biofilm layer, injected microbes gain additional opportunities to grow and dominate the population. These results theoretically showcase how metabolic interactions contribute to the growth, distribution, abundance, stability, and integrity of gut biofilm structures.

## Methods

### Model overview

The work aimed to simulate the attachment and growth of bacteria within a biofilm, in order to predict its population and structural dynamics. Bacterial size and position capture physical constraints on populations, while diffusion gradients and bacterial feeding simulated chemical interactions. The combined model simulated the growth and interactions that generated a biofilm ab initio within arbitrary topologies and nutritional environments. Through simulation, we predicted the macroscale properties of biofilms within complex environments, including surface patterning, dominant species, and bulk populations after growth.

### ABM description

Coupled ABM and FVM simulations were used to simulate interactions between bacteria and arbitrary nutrients and metabolites. Based on previously validated biofilm ABM models^28,29^, the ABM simulated bacteria as spherical individual agents with a continuous position in a 2D box (500 μm × 500 μm), allowing for realistic movement and replication. Bacterial agents were assigned a mass and radius, as well as state variables representing their attachment to a biofilm. They were assumed to be from one of a fixed number of predetermined species, and all members of the same species had the same growth rates and feeding behavior within the simulation. We simulated the evolution of position, size, and quantity of bacteria over hours to days. Collision constraints were imposed to prevent bacteria from intersecting with substratum boundaries or each other, and movement was assumed to be inertia-free due to the low Reynolds number of microscale environments.

A lattice (50 × 50) discretized the solid substratum into (10 μm × 10 μm) blocks. This allows for arbitrary substratum shapes, out of which we chose a corrugated crypt-like typography (pillar height = 80 μm, pillar diameter = 20 μm, pillar pacing = 30 μm) for our experiments. The fluidic environment of the biofilm was simulated on the same lattice using the FVM to solve for the reaction-diffusion equations of chemical species. It maintained periodic boundary conditions for vertical side walls, no-flux boundary conditions on substratum boundaries, and fixed concentrations on the top surface, simulating a bulk fluid. FVM is a popular choice for fluid modeling, with the FiPy package providing an efficient implementation that interoperates easily with our NumPy and Python-based simulation^37^. Solutes diffused from the bulk fluid to the surface of the substratum, fueling biofilm growth. The growth cycle of the biofilm began with an initial attachment period where planktonic cells freely adhered to the substratum surface. Thereafter, bacteria grow, replicate, and form colonies.

### Process overview

The ABM simulation iterated in timesteps, in which movement, shoving, growth, and replication are simulated sequentially. A single timestep represented up to 15 min of real-time growth, with shorter timesteps taken for the planktonic movement phase of simulations (see Supplementary Fig. 15 for the model flow chart, Supplementary Table 1 for the list of simulation parameters, their definitions, and values. Also, see the ABM Code Implementation section in the SI for a detailed description of the code modules and their interactions).

#### Planktonic movement

To simulate bacterial planktonic attachment, bacteria entered the field at *t* = 0 randomly placed in the spacing above the host substratum, at 200 μm above pillars, and below the upper boundary. The bacteria were allowed to interact with the substratum using a run-and-tumble motility model, approximated by displacements consisting of translational (10 μm/s) and rotational components (<*θ*> = 45°) during each time step (1 s), consistent with the ranges reported in the literature^79,80^. Bacteria making contact with the surface or other attached bacteria were marked as biofilm members and cease movement. The planktonic attachment phase lasts 100 s with 200 bacteria (~10^9^ bacteria/mL, for a depth of the field of 1 μm^42,81^), during which bacteria adhered to the host substratum in sufficient numbers to simulate a precursor biofilm layer. Bacterial detachment was ignored, and biomass accumulation, following the same metabolic rules as other times, was minimal during this stage. After this initial attachment phase, the biofilm growth continued in 15-min increments. For simulations employing newcomer invasion, newcomers (100 bacteria), again randomly distributed, enter the field just above the biofilm surface (200 μm above the pillar top surface) and below the top boundary for a duration of 100 s.

#### Mechanical interactions in the biofilm

As bacteria grow or replicate, space limitations cause some cells to overlap, while solid surfaces such as pillars constrain their position. To address this issue, a shoving algorithm, similar to that previously used by Lardon et al., was incorporated^29^, interspersed with a wall constraint algorithm to prevent overlap with a solid surface.

At each shoving step, bacterial cells within a biofilm were selected, and center-to-center distances between each cell and each overlapping cell were calculated. Pairs of displacement vectors with a length equal to half of the distance and along the cell center-to-center direction were defined for intersecting cells. Candidate updated positions were calculated in parallel for each cell at once by shifting each cell position by the sum of their displacement vector calculated against every overlapping cell.

In the wall constraint update, bacteria were moved along the distance from their current position to their candidate new position. If they would intersect with a substratum surface along the way, movement was halted at the point where they would first make contact. Bacteria positions were updated to the first point of contact or their candidate position if no contact is made.

The shoving and wall constraint algorithms are applied to all the cells in parallel, and applied for multiple iterations at each time step, ensuring spatial separation and wall constraints among all cells.

#### Bacterial metabolism and growth

Bacterial metabolism follows the Monod kinetic expression:

1 $$\mu ={\mu }_{\max }\frac{S}{{K}_{s}+S}$$

where *S* is the nutrient concentration, ${\mu }_{\max }$ is the maximum specific growth rate, and ${K}_{s}$ is the saturation constant. For the base model, ${\mu }_{\max }$ was taken as 0.3 h^−1^ and *K*_*s*_ as 0.2 g/L for bulk nutrients based on the range of reported estimates^82,83^. The biomass changes and nutrient consumption for each bacterium in each timestep were calculated from:

2 $$\frac{{dx}}{{dt}}=(\mu -m)x$$

3 $$\frac{{dS}}{{dt}}=\frac{-1}{{Y}_{x/S}}\mu x$$

where *x* is the biomass, *m* is the specific cell maintenance coefficient, and *Y*_*x/s*_ is the yield coefficient*. Y*_*x/s*_ and *m* were set to 0.15 (dimensionless) and 0.03 (h^−1^), respectively, for all the nutrient sources approximating the published data^82,84^. Having solved the reaction-diffusion equation in each grid block (see below), growth for each bacterium was computed during each timestep and mass was incremented by the product of growth rate and time step. If the initial average cell masses are taken as 1 unit of mass, cells divided when they reach an average mass of 2. Both the initial cell masses and the threshold mass at which they divide were set to have a Gaussian distribution with 10% Coefficient of Variation and cut off outside two standard deviations^29^. The daughter cell was positioned in a random direction tangent to the parent cell.

#### Diffusion of chemical species

The model solved for the concentrations of chemical species using FVM through the FiPy Python package^85^. Considering the normally slow flow regime in the large intestine, a Fickian diffusion with a diffusion coefficient (*D*) was approximated for the mass transport of bulk nutrients and metabolic by-products. Monod kinetics were adopted for the reaction rates^39^. A pseudo steady state assumption for chemical diffusion with respect to biofilm growth was made to compute the temporal variation of chemical species, akin to previous works^45^. For each timestep, the equation to solve the concentration of the chemical species, including bulk nutrients or produced metabolites (*S*), at each location represented by the position vector (*X*) was as follows:

4 $$\nabla .\left[{D}_{s}\left(X\right).\nabla S\left(X\right)\right]+{R}_{s}\left(X\right)=0$$

The values of *D* for nutrients and metabolites are listed in Supplementary Table 1. *D* inside the biofilm matrix was assumed to be 0.8 of that in the bulk fluid, according to other works^29^. We set a bulk value (*S*_*b*_) of 4 C-mmol/L (glucose equivalent) at the top boundary close to the concentration used in gut ABM models^86^. No-flux condition was enforced on the substratum. For the models simulating the host effects, the host metabolite concentration was set constant at the substratum surface. *R*_*S*_*(x)* was approximated as a constant within each grid block during each time step and calculated as the product of bacteria consumption per unit mass given by the Monod equation and bacterial mass within the cell estimated by summing the mass of each agent inside. While solving for chemical concentrations, bacterial concentration was assumed to change so slowly as to be effectively constant. For a bulk nutrient or metabolite *S*_*i*_, with concentrations of each biomass type *x*_*k*_ (*k* = 1, 2, …, *m*) in point *X*, giving the equation:

5 $$\begin{array}{l}{R}_{{Si}}\left(x\right)=-\mathop{\sum}\limits_{k=1}^{m}{\mu }_{\max \left(k\right)}\frac{{S}_{i}}{{S}_{i}+{K}_{s\left(i,k\right)}}{x}_{k}{Y}_{{S}_{i}/{x}_{k}}\\\qquad\qquad\,\,\,+\,\mathop{\sum}\limits_{k=1}^{m}\mathop{\sum}\limits_{j=1}^{n}{\mu }_{\max (k)}\frac{{S}_{j}}{{S}_{j}+{K}_{s(j,k)}}{x}_{k}{Y}_{{s}_{i}{/x}_{k}}\end{array}$$

Note that the first term refers to the consumption of a bulk nutrient or a metabolic by-product *S*_*i*_, and the second term refers to the generation of *S*_*i*_ from the cellular consumption of bulk nutrients or other metabolic by-products *S*_*j*_ (*j* = 1,2, …, *n*).

#### Segregation index

The segregation index was calculated using a Euclidean distance-based K-nearest neighbor algorithm (leaf size = 2, number of neighbors = 8). This method provides a reliable measure of segregation—for further discussion refer to the Supplementary Information (Supplementary Simulations and Supplementary Fig. 16). For each bacterium, the ratio of neighboring bacteria of the same type was taken as the segregation index. The segregation for a multispecies community was defined as a matrix with each element (*i*,*j*) corresponding to the number of bacteria of type *j* neighboring bacteria of type *i*.

### The ODE model

The differential equation model computes the temporal change of the bacterial population, assuming spatial heterogeneity in the biofilm. It solves the following system of equations, accounting for mass balances for each biomass type (*x*_*k*_) and chemical species (*S*_*i*_):

6 $$\frac{d{x}_{k}}{{dt}}=\left(\mathop{\sum}\limits_{i=1}^{l}{\mu }_{\max }\frac{{S}_{i}}{{S}_{i}+{K}_{s}(i,k)}-m\right){x}_{k}$$

where bacterium k uptakes substrates *S*_*i*_ (*i* = 1,2, …, *l*).

7 $$\begin{array}{l}\frac{d{S}_{i}}{{dt}}=\frac{K}{{V}_{{biofilm}}}\left({S}_{b}-{S}_{i}\right)-\mathop{\sum}\limits_{k=1}^{m}{\mu}_{\max}\frac{{S}_{i}}{{S}_{i}+{K}_{s(i,k)}}{x}_{k}{Y}_{{S}_{i}/{x}_{k}}\\\quad\quad\,\,\,\,+\,\mathop{\sum}\limits_{k=1}^{m}\mathop{\sum}\limits_{j=1}^{n}{\mu }_{\max }\frac{{S}_{j}}{{S}_{j}+{K}_{s(j,k)}}{x}_{k}{Y}_{{S}_{i}{/x}_{k}}\,\end{array}$$

where *K* refers to the mass transfer coefficient across the diffusional layer (*K* = $\frac{{DA}}{L}$; ${V}_{{biofilm}}=A{L}^{{\prime} },\,$*D*: diffusion coefficient; *A*: cross-section of the field, (500 μm × 1 μm); *L*: diffusion length, 400 μm; *L’*: biofilm height = 200 μm). Note that, in Eq. (6), the term ∑ refers to *S*_*i*_ consumption as a bulk nutrient or a metabolic by-product, a sink term with kinetics depending on *S*_*i*_. The term ∑∑ refers to *S*_*i*_ generation as a metabolic by-product from nutrients or other metabolic by-products (*S*_*j*_), a source term with kinetics depending on *S*_*j*_. The initial total biofilm biomass was equal to the mass of 200 bacteria and the kinetic parameters were the same as ABM simulations.

### Reporting summary

Further information on research design is available in the Nature Research Reporting Summary linked to this article.

## Supplementary information

Supplementary Information

Reporting summary

## Data Availability

Raw data generated for the current study are available from the corresponding author.

## Code availability

Codes used for the current study are available from the corresponding author.
