# Supplementary material for: Metabolic interactions shape emergent biofilm structures in a conceptual model of gut mucosal bacterial communities
Source: NPJ Biofilms Microbiomes. 2024 Oct 2;10:99. doi: 10.1038/s41522-024-00572-y (PMC11447261; doi:10.1038/s41522-024-00572-y)
Supplement: Supplementary file 1 — Supplementary Information [file 41522_2024_572_MOESM1_ESM.pdf]

# Supplementary Information

## Metabolic interactions shape emergent biofilm structures in a conceptual model of gut mucosal bacterial communities

Amin Valiei[1], Andrew Dickson[1], Javad Aminian-Dehkordi[1], Mohammad R.K. Mofrad[1,2*]

1. Molecular Cell Biomechanics Laboratory, Departments of Bioengineering and Mechanical Engineering, University of California, Berkeley, California 94720, USA

2. Molecular Biophysics and Integrative Bioimaging Division, Lawrence Berkeley National Lab, Berkeley, California 94720, USA

*Corresponding author: mofrad@berkeley.edu

# I. SUPPLEMENTARY SIMULATIONS

**Modeling biofilms with oxygen-dependant kinetics**

In this case study, we investigated the influence of the oxygen gradient near the host surface on mucosal biofilm structures. The variation of oxygen across the mucosa can impact bacterial growth in this region due to the correlation between the growth rate and oxygen level. In a proof-of-concept investigation, we estimated how this effect unfolds for various metabolic interaction types. Various models exist for describing growth dependence on oxygen. In these models, aerobic and anaerobic bacteria show different kinetic behaviors. For example, two models used for strict anaerobic (Equation 1) and facultative aerobic bacteria (Equation 2) are[1]:

$$\mu'_{max} = \mu_{max}\left(1 - \frac{\%O_2}{MIC}\right)^{\alpha} \qquad \text{(Equation 1)}$$

$$\mu'_{max} = \mu_{max}\left(\frac{\%O_2}{K_s + \%O_2}\right) \qquad \text{(Equation 2)}$$

where %O2 is the oxygen concentration (in the gaseous phase), $\mu_{max}$ is the maximum specific growth rate, *MIC* is the minimum inhibitory concentration, $\alpha$ *is* the curve ratio, and $K_s$ is the saturation constant. For this preliminary study, we compared the patterns developing from two bacterial types, a strict anaerobe, labeled type "a", and a facultative aerobe, labeled type "b." We set $\mu_{max\,(a)}$ to 0.4 $h^{-1}$ and $\mu_{max\,(b)}$ to 0.6 $h^{-1}$ (50% larger for the latter). We set *MIC* to 6.61 and $\alpha$ to 0.76 for type "a" and $K_s$ (oxygen) to 0.12% for type "b." These were taken from a study on multiple relevant aerobic and anaerobic strains[1].

The oxygen content at the host surface (3% ~ 22 mm Hg) is higher than the lumen (1.5% ~ 11 mm Hg)[2,3], conditions that are associated with the coexistence of anaerobic and aerobic bacteria, notable in the ileocecal region and initial segments of the colon[2,3]. These, respectively, constitute the boundary conditions for oxygen concentration at the substratum and the top boundary in the simulation. In Supplementary Equations 1 and 2, variation of the oxygen concentration slightly impacts the growth rate of type "b" but more considerably impacts the growth of type "a". We obtained results for different metabolic interaction types for three scenarios of oxygen kinetics: (i) both bacteria are type "a" indicating that their kinetics described by Supplementary Equation 1; (ii) one bacterium is type "a" described by Supplementary Equation 1 and the other is type "b" following Supplementary equation 2; and (iii) both bacteria are type "b" described by Supplementary Equation 2.

Results shown in Supplementary Figure 7 illustrated that bacteria with the same oxygen kinetics (cases i and iii) tend to produce similar morphological and populational patterns observed in the simulations without considering oxygen kinetics (Figure 2). In case ii, where the kinetics were different, competition resulted in the dominance of the bacterium “b”, having the fastest growth rate. Mutualism featured the slightest variation in populational and structural properties (Supplementary Figure 7).

**Segregation Index**

The concept of the segregation index for microbial community models in which agents have continuous coordinates has been previously employed in other studies[6,7].

Based on our analysis, both the nearest-neighbors and distance-based criteria yield similar results for the calculation of the segregation index, providing robust predictions.

In Supplementary Figure 16, we show the results from calculating the segregation index for the baseline competition scenario for brown bacteria using k=8 nearest neighbors and *k*=20 nearest neighbors, each with a cut-off distance of 20 μm, and with no cut-off distance. In these calculations, the cut-off distance refers to the radius within which the cell types are analyzed. As depicted in the graph, the results are similar and consistent across all cases. In this study, we selected nearest-neighbors method with k=8 and without any cut-off distance assumptions.

## II. SUPPLEMENTARY FIGURES

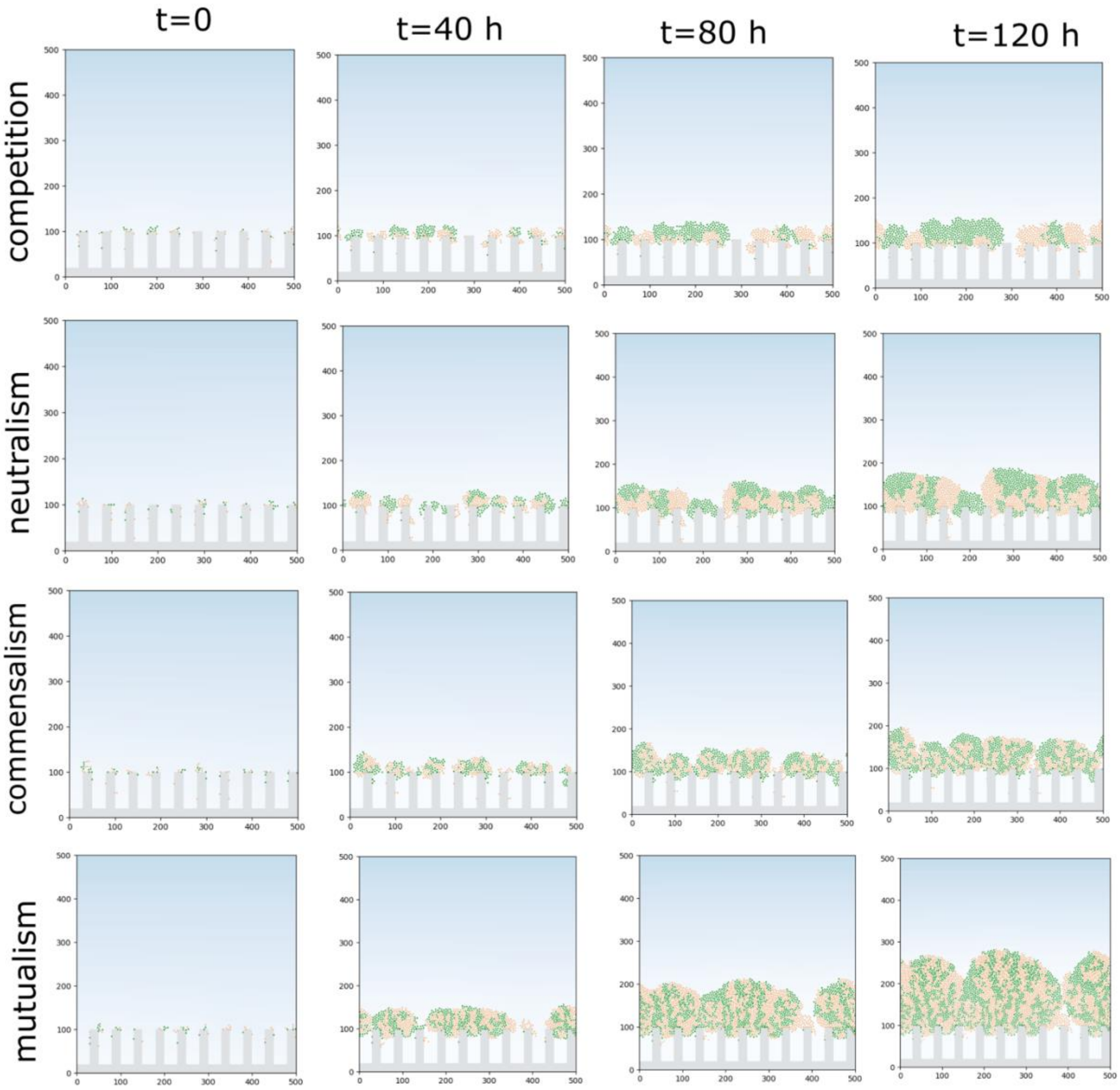


**Supplementary Figure 1.** Temporal evolution of biofilm morphologies from different types of metabolic interactions.

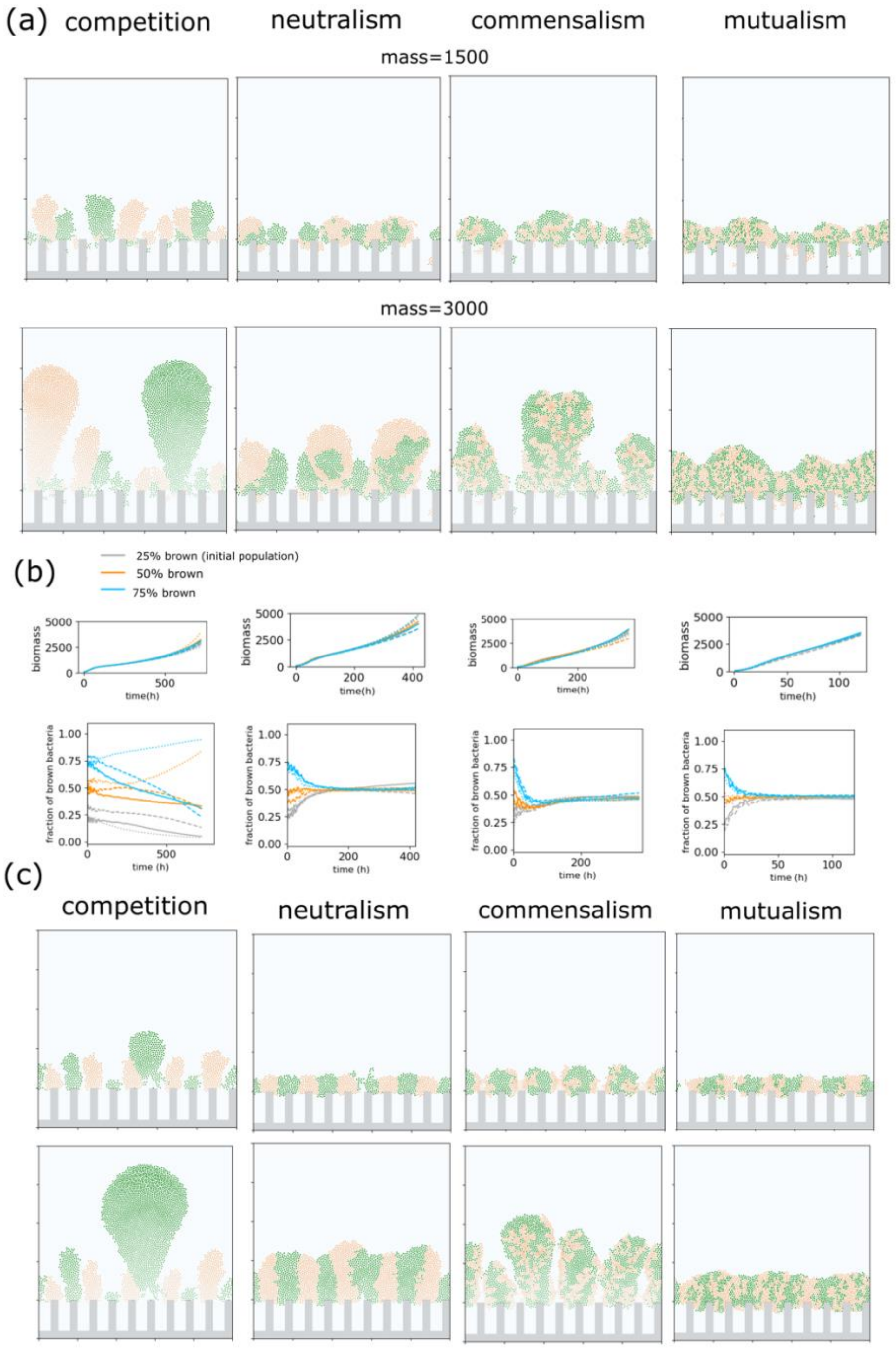


**Supplementary Figure 2.** Comparison of biofilm structures having equal amounts of biomass. **(a)** The first row shows biofilm structures resulting from each interaction type at an equal biomass amount of 1500. The second row shows the structure at a biomass amount of 3000. The unit of mass is the mass of a bacterium with a diameter of 1 μm. **(b)** Populational features of biofilms, including the amount of total biomass and the fraction of brown bacteria with time. **(c)** Biofilm structures resulting from a sequential attachment pattern (one bacteria on the center of each pillar in an alternating pattern). The top row shows the results at the mass of 1500 and the bottom at the mass of 3000. The biomass unit is the mass of a bacterium with a diameter of 1 μm.

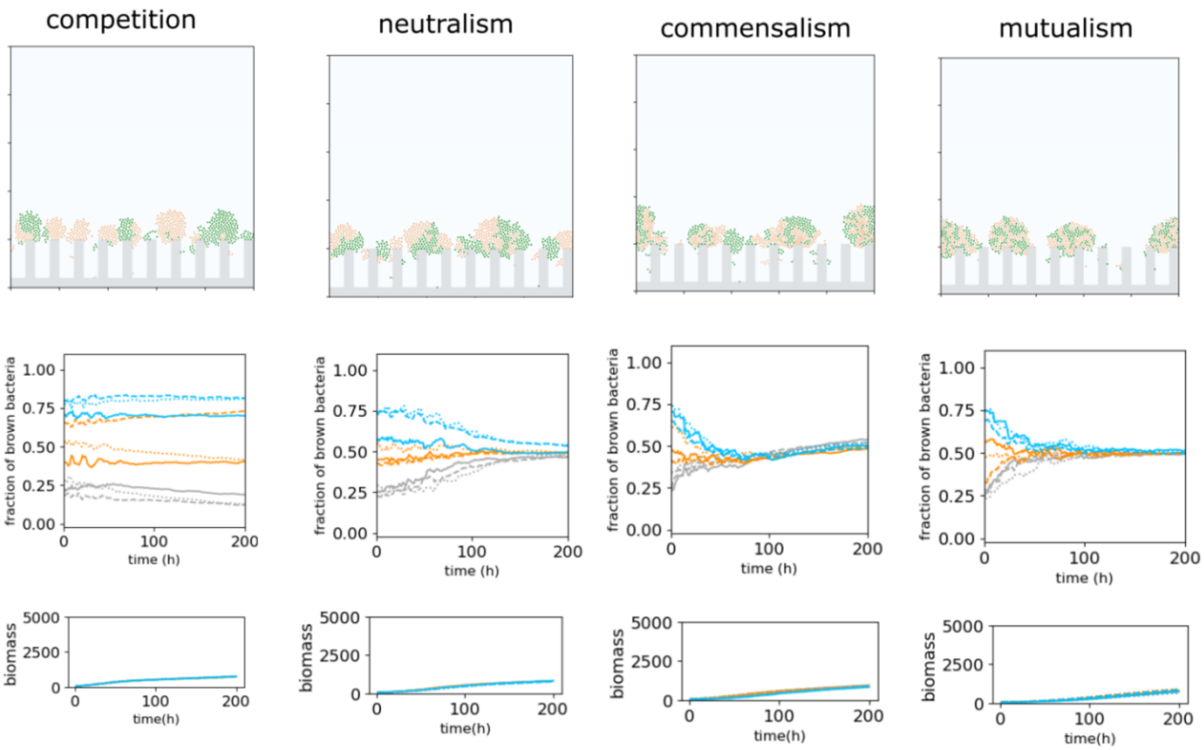


**Supplementary Figure 3.** Comparison of biofilm morphology and bacterial population resulting from various types of metabolic interactions. In this simulation, the maximum specific growth rate of bacteria in each scenario ($\mu_{max}$) was scaled such that biofilms have identical mass at *t*=200 h (scale factors: competition: 1, neutralism: ~0.9, commensalism: ~0.8, mutualism: ~0.5). We observe distinct morphologies highlighting segregated arrangement in competition and neutralism and intermixed patterns in commensalism and mutualism.

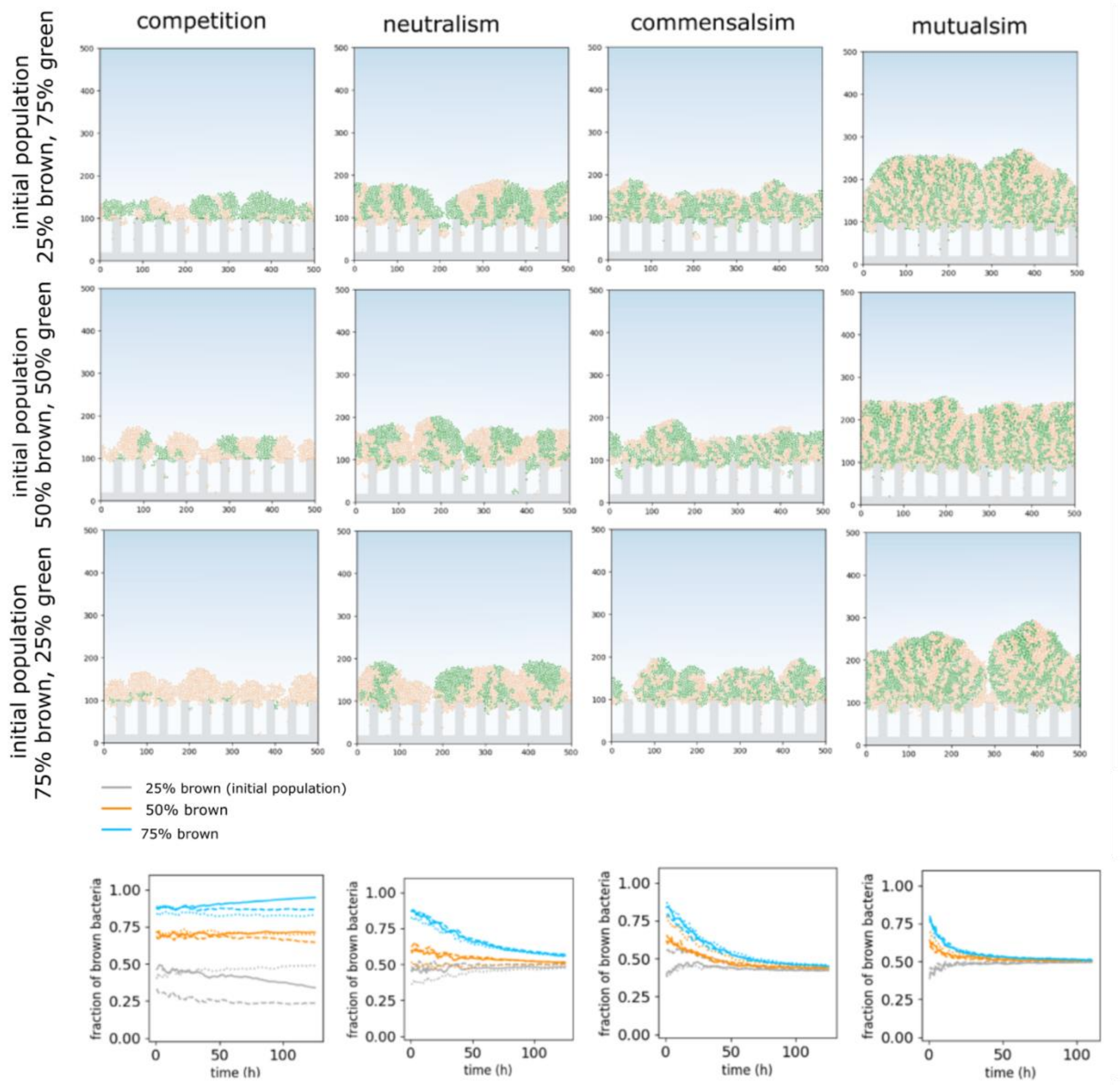


**Supplementary Figure 4.** Effect of bacterial motility on biofilm growth. In this set of simulations, the motility of brown bacteria (20 μm/s) is twice that of green bacteria (10 μm/s)—the rest of the parameters are the same as the baseline simulation. This causes a higher initial attachment for brown bacteria; however, the morphological and populational patterns of the biofilm remain the same as the baseline simulation (snapshots are at $t$=120 h). Each line type represents a repetition, and each line color corresponds to an initial population composition.

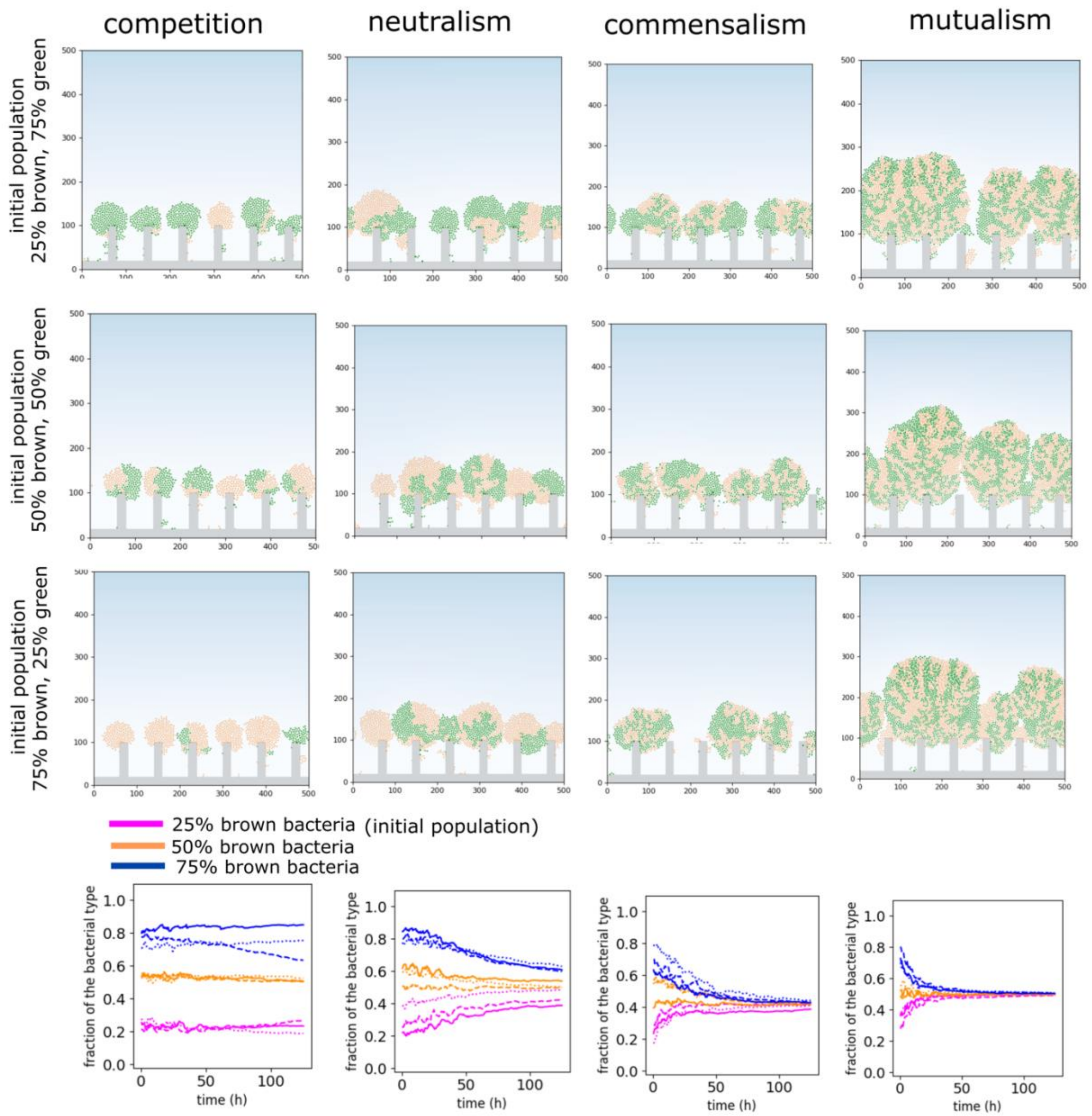


**Supplementary Figure 5.** Biofilm qualitative and quantitative properties in a simulation with crypt spacing 60 μm. Biofilm architecture is slightly different due to different attachment patterns (snapshots are at *t*=120 h). Relative abundances are similar to the baseline case, with a spacing of 30 μm. Simulation paratmers for each bacterium are the same as the baseline case. Each line type represents a repetition, and each line color corresponds to an initial population composition.

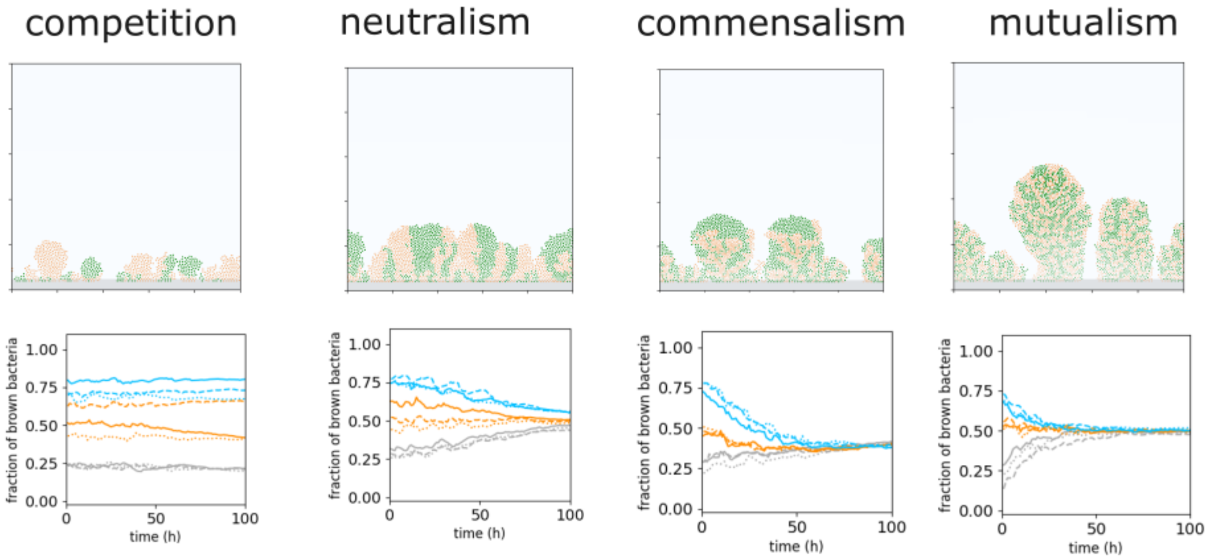


**Supplementary Figure 6.** Distinct biofilm morphologies and relative abundances emerge from different types of interactions on a flat topography. The top row shows biofilm structures at *t*=120. All simulation parameters and assumptions remained consistent (the same as the baseline scenario shown in Figure 2), except for the alteration to the substratum topography. Biofilms in competition and neutralism scenarios consist of separate patches contrasting with intermixed populations seen in commensalism and mutualism.

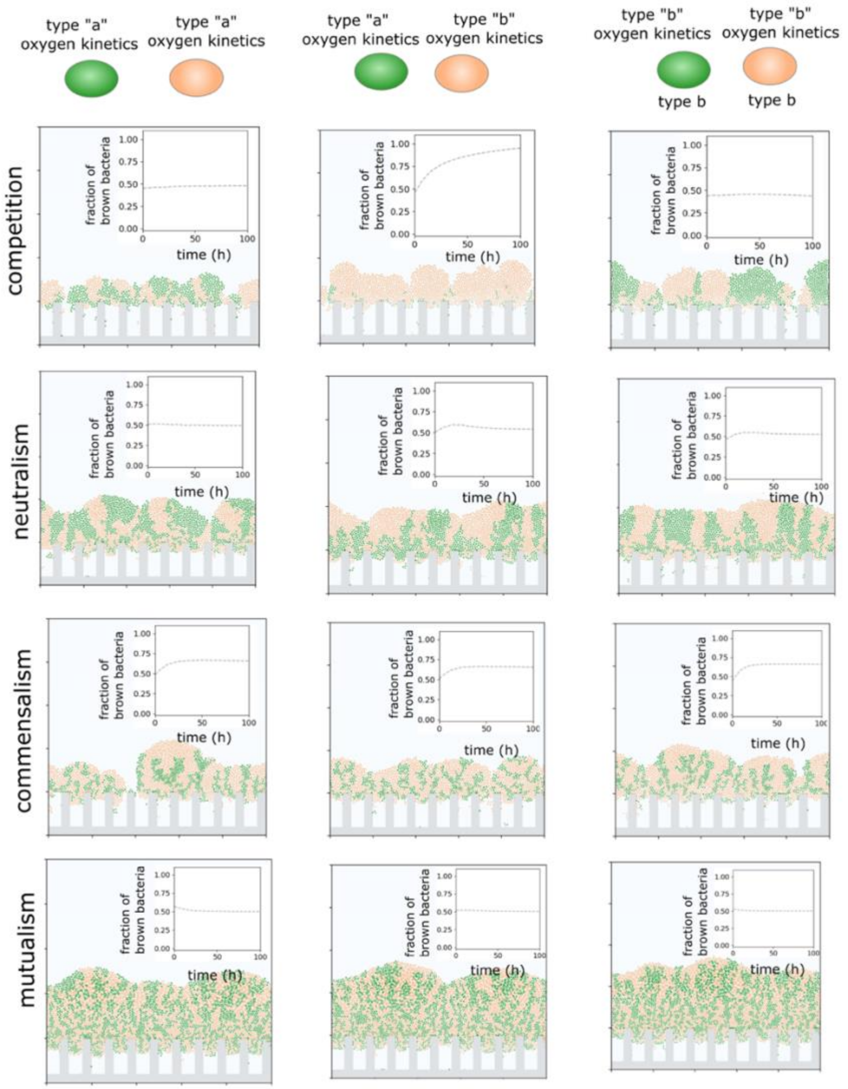


**Supplementary Figure 7**. Morphological and populational variations among metabolic interaction scenarios with the inclusion of an oxygen gradient along the crypt axis. The results encompass different scenarios of the combination of type "a" and type "b" kinetics as described above. Type "b" has an unmodified maximum specific growth rate ($\mu_{max}$) 50% higher than those of type "a"[1]. Oxygen was allowed to diffuse from the host surface into the microbial biofilm. Oxygen concentration was assumed constant (3% ~ 22 mm HG) at the host surface[2]. The kinetics of oxygen dependency are governed by Supplementary Equations 1 and 2. Snapshots were taken at *t*=100 h.

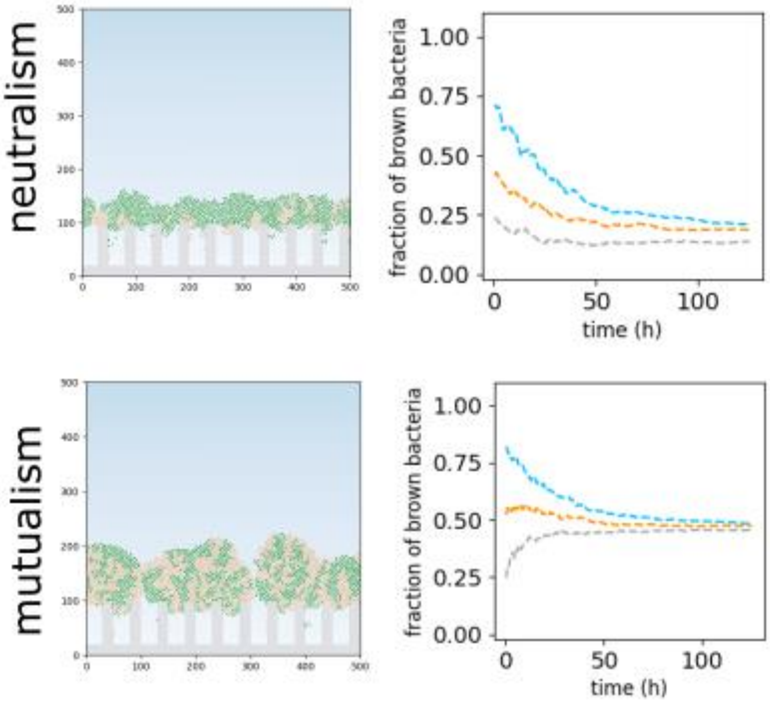


**Supplementary Figure 8.** Effect of substrate concentration on the relative abundance for the neutralism and mutualism metabolic interaction scenarios. In this simulation, the concentration of the bulk nutrient the green bacteria are feeding on was increased by 50% (6 C-mmol/L). Snapshots are taken at *t*=120 h and the simulation parameters for each bacterium is the same as the baseline simulation. Mutualism is more robust against perturbations in the concentration of nutrients.

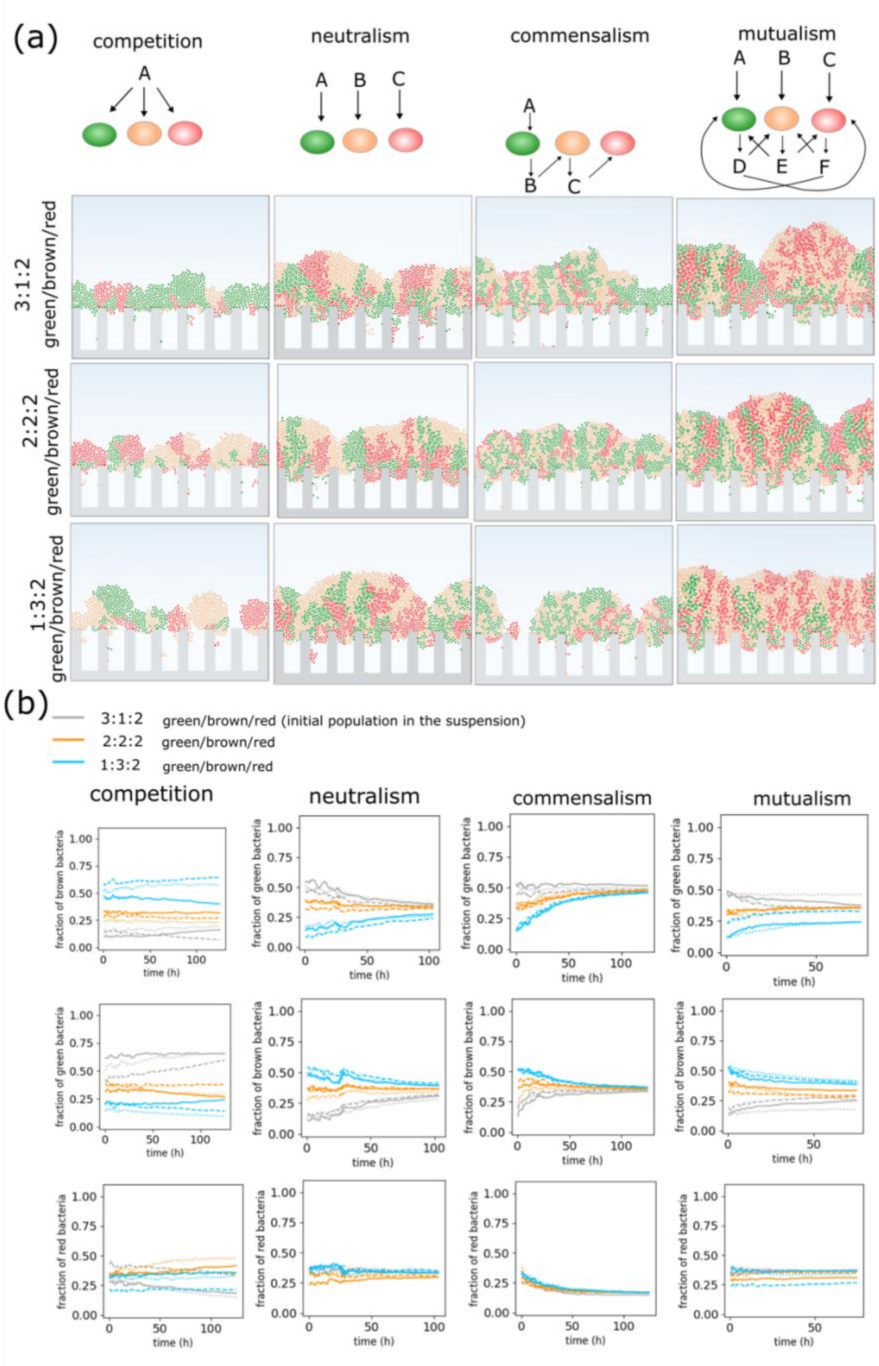


**Supplementary Figure 9.** Properties of three-species biofilms in various metabolic interaction scenarios with different initial relative abundances. This scenario is the expansion of the baseline simulation (Figure 2) to three-species communities. The total number of injected bacteria in the suspension in all the simulations is 300 (200 brown and green bacteria and 100 red bacteria), and the simulation parameters for each bacterium are the same as the baseline simulation (Figure 2)—for a list of simulation parameters refer to Supplementary Table 1. **(a)** The morphology of biofilms shows the fundamental characteristics seen in two-species biofilms are preserved. **(b)** Quantitative features also aligne with the fundamental characteristics seen in two-species biofilms. Each line type represents a repetition, and each line color corresponds to an initial population composition.

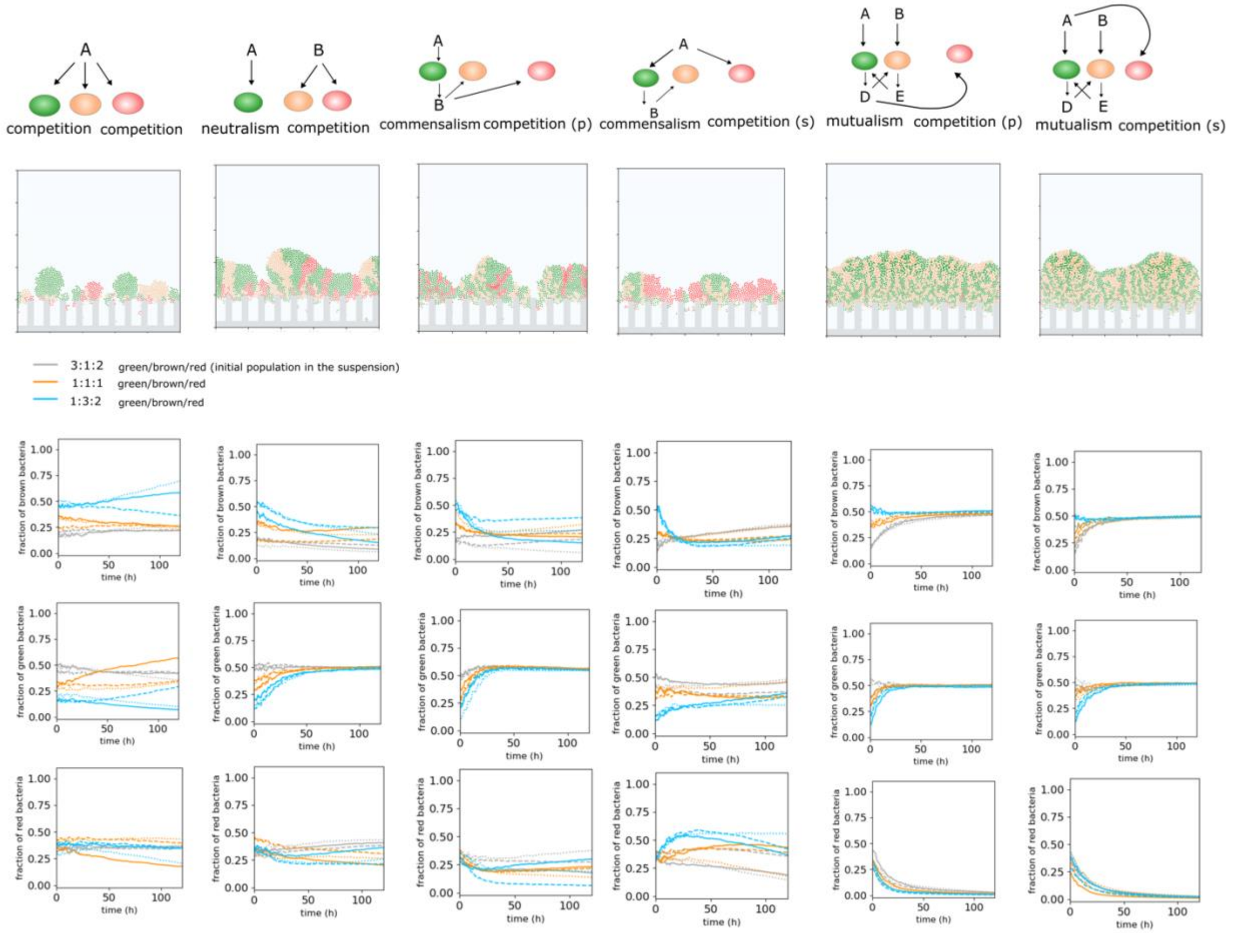


**Supplementary Figure 10.** Quantitative and qualitative biofilm characterization for three-species biofilms in a cocolonization scenario. The invading strain (red), which is in competition with existing bacteria according to the top schematics, is able to grow in competition, neutralism, and commensalism but not in mutualism. (p) refers to the competition on metabolic by-products produced by bacteria, and (s) refers to the competition on nutrients diffusing from the bulk fluid. The snapshots correspond to the case study with an equal relative abundance of all bacterial types. In all these simulations, the total number of planktonic bacteria is 300 (green and brown: 200 and red:100), and the simulation parameters are the same as the baseline simulation for all bacteria (refer to the Methods and Supplementary Table 1). Snapshots are taken at *t*=120 h. Each line type represents a repetition, and each line color corresponds to an initial population composition.-

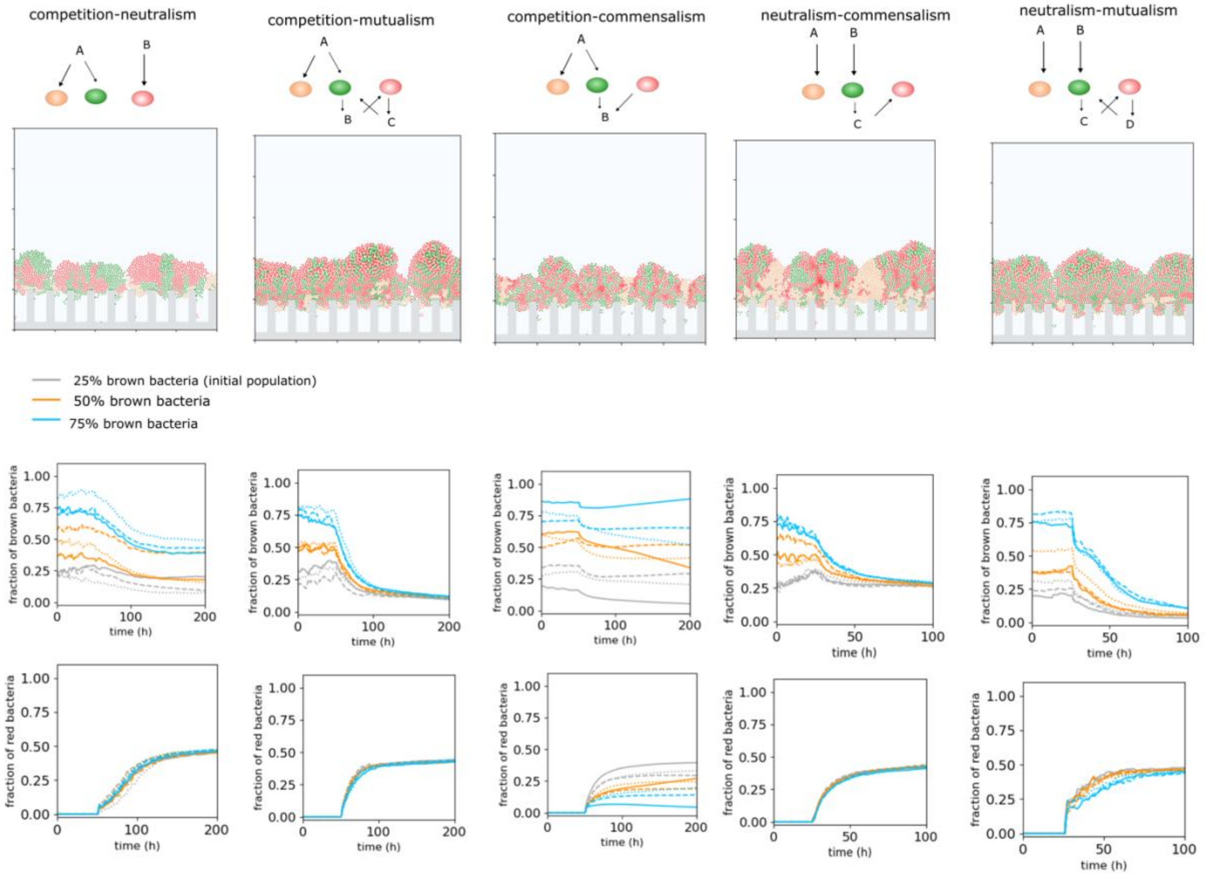


**Supplementary Figure 11.** Biofilm morphology and composition resulting from the invasion of red bacteria engaging in neutralistic, commensal, or mutualistic interactions with an existing biofilm of green and brown bacteria according to the top schematics. Invasion occurs at *t*=25 h, and the snapshots are taken at *t*=200. The kinetic parameters of bacteria were set equal to those of the baseline simulation (Figure 2)—refer to Supplementary Table 1 for a list of simulation parameters. The bacterial count in the initial population is 200 and count of invading bacteria is 100. When red bacteria have cooperative (commensalism, mutualism) relations with the existing biofilm, the invading species intersperse within the biofilm structure.

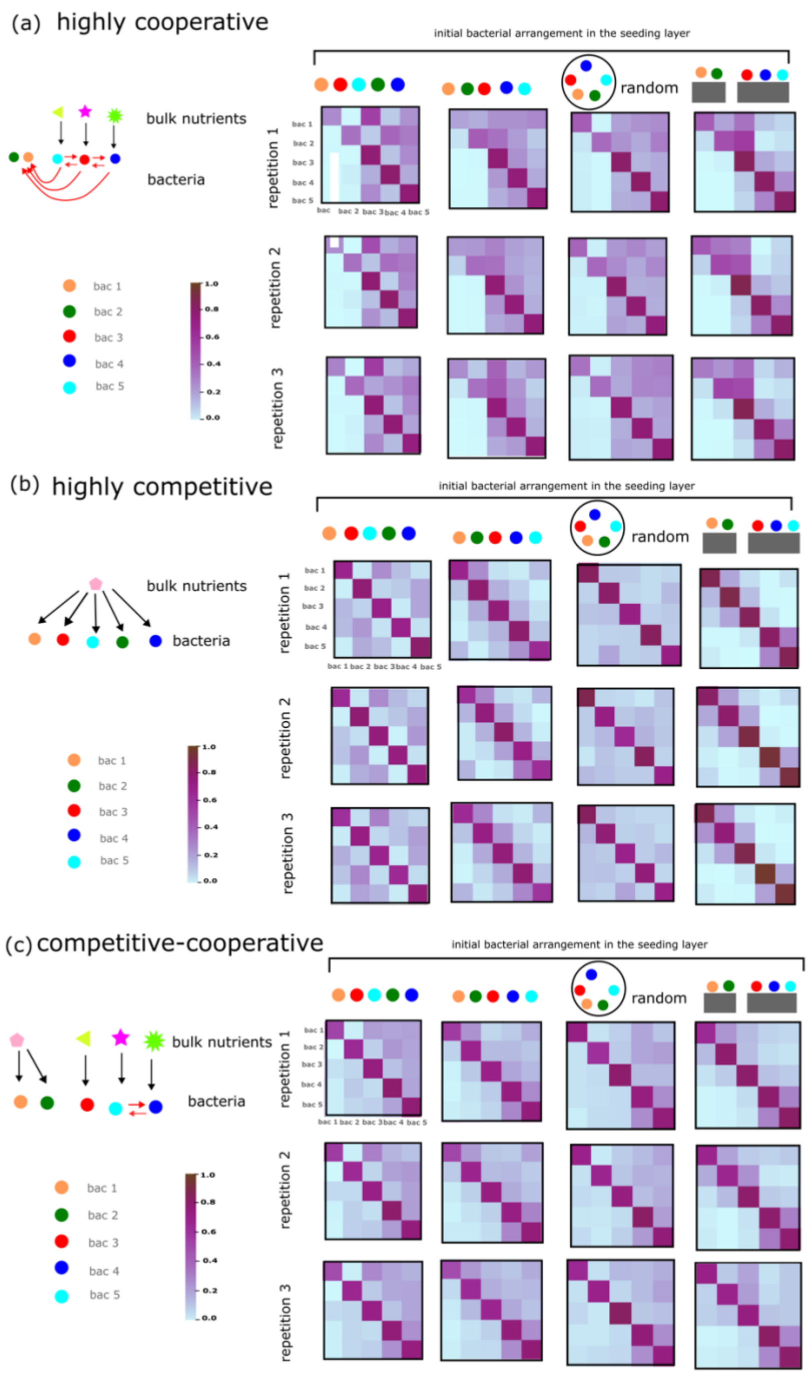


**Supplementary Figure 12.** Segregation index matrix (refer to the Methods) for polymicrobial biofilms. **(a)** The segregation index matrix is plotted as heat maps for highly cooperative interaction among bacteria. As the legend shows, three bacteria (cyan, blue, and red) are engaged in mutualism, while two bacteria (brown and green) are involved in commensalism with the mutualistic bacteria. The schematic above each column shows the initial arrangement of bacteria. The heat plot shows the consistency of the outcome for each repetition and its dependence on the initial attachment pattern. **(b)** The segregation index calculation for a highly competitive regime. In this scenario, all the bacteria compete with each other over the same bulk nutrient. **(c)** The segregation heat maps for a composite cooperative-competitive scenario where blue and cyan bacteria are involved in mutualism, green and brown interact through competition, and red bacteria have a neutralistic relationship with others.

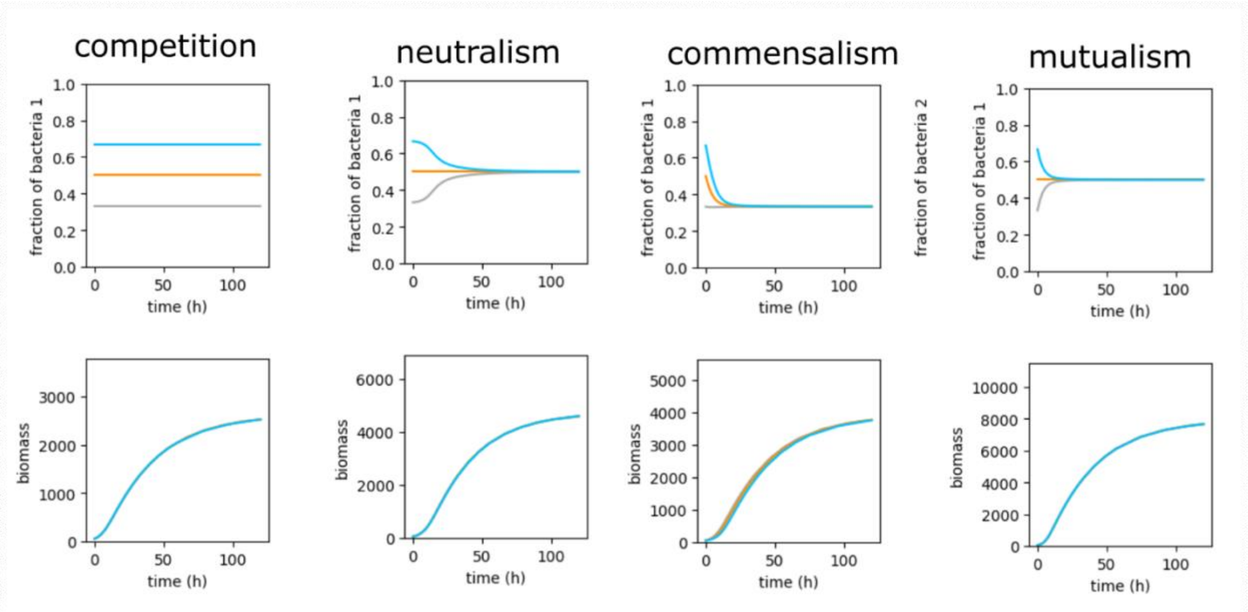


**Supplementary Figure 13.** Biomass growth and species abundance in a dual-species biofilm community, computed from the ODE model.

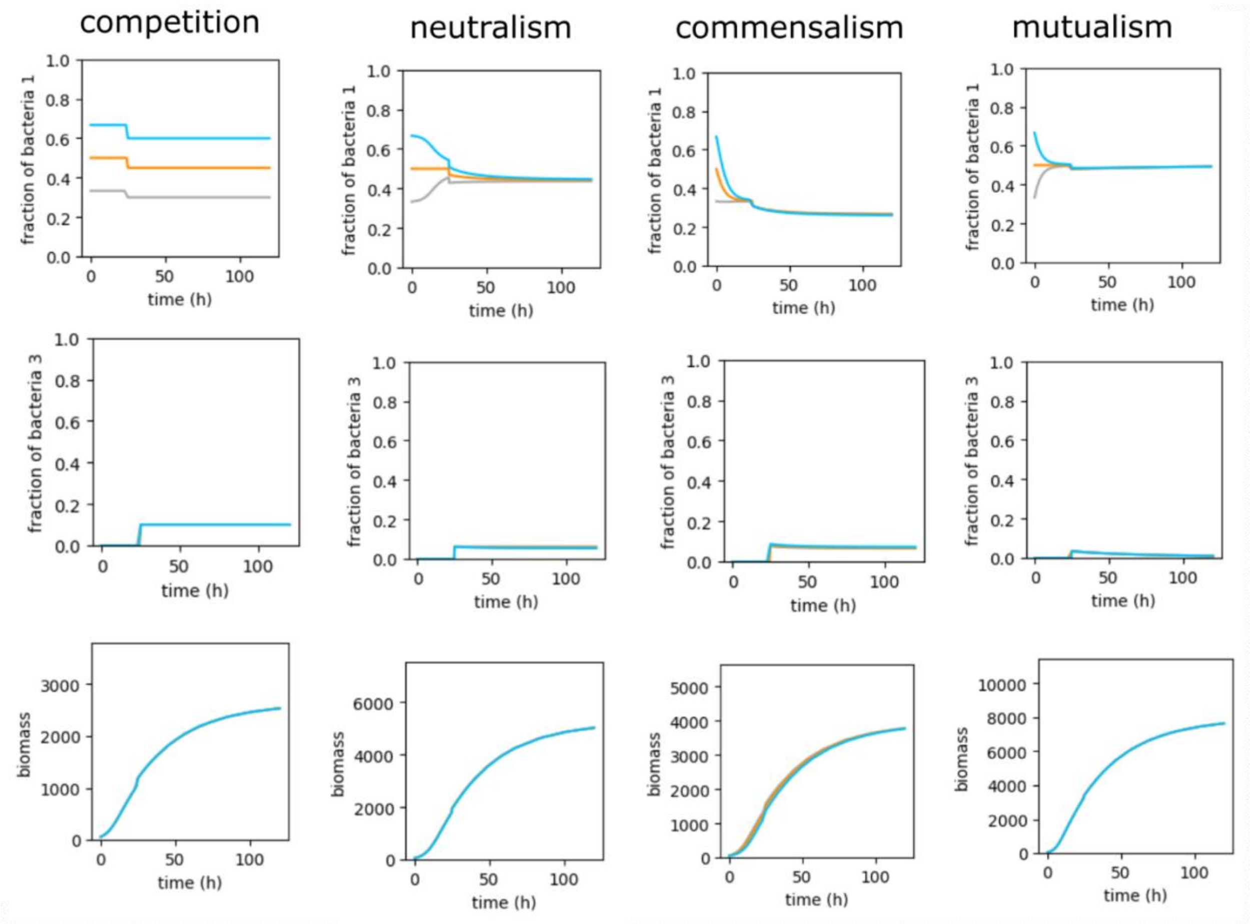


**Supplementary Figure 14.** Biomass growth and species abundance in a dual-species community obtained from the ODE model.

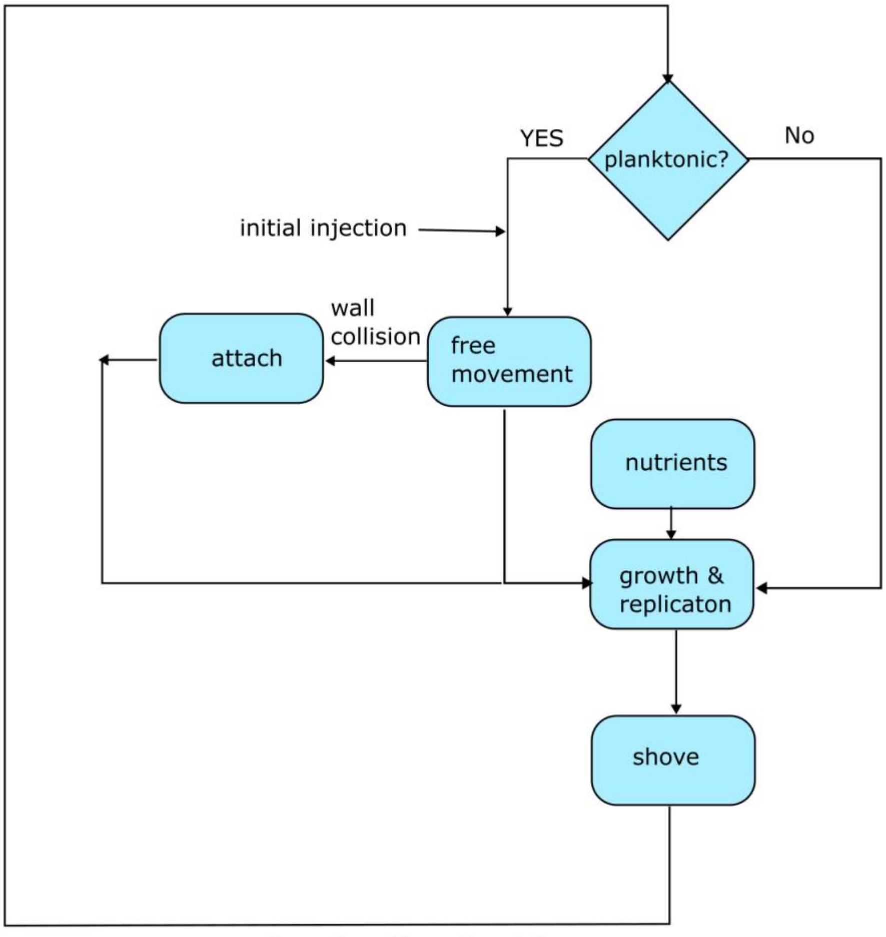


**Supplementary Figure 15.** Flowchart showing the overall ABM rule for agents (bacteria).

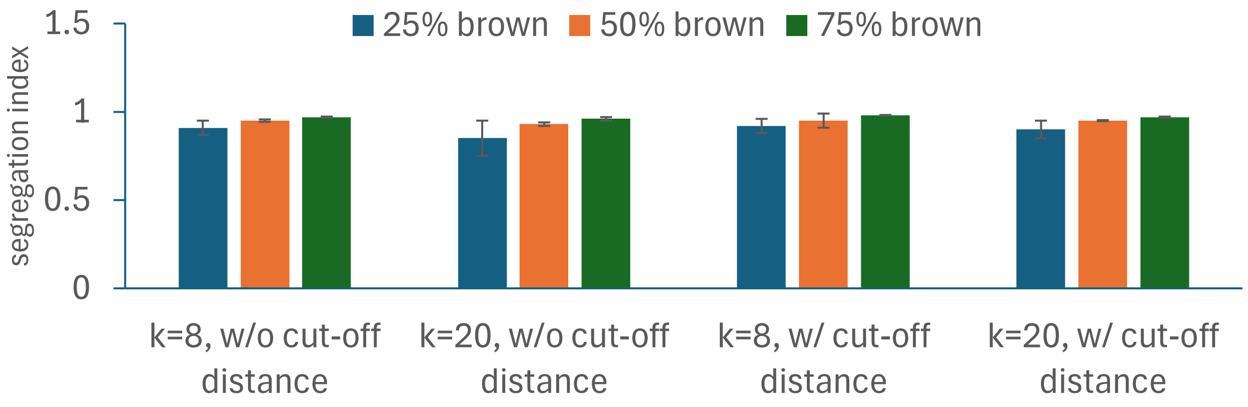


**Supplementary Figure 16.** The values of the segregation index calculated for the baseline competition scenario for various definitions explained in the Supplementary Simulations. All the calculations are based on the k nearest neighbor method, including scenarios with k=8 and k=20. The simulations also include scenarios based on defining or not defining a cutoff distance. Error bars represent the standard deviations.

# III. SUPPLEMENTARY TABLE

**Supplementary Table 1.** Physicochemical parameters used in the model.

| Parameter definition | **Symbol** | **Unit** | **Value** |
|---|---|---|---|
| Saturation constant | $K_s$ | $g.L^{-1}$ | 0.2 (ref[5]) |
| Maximum specific growth rate | $\mu_{max}^{*}$ | $h^{-1}$ | 0.3 (ref[8]) |
| Maintenance rate | $m$ | $h^{-1}$ | 0.03 (ref[9]) |
| Biomass yield coefficient | $Y_{x/s}$ | $C\text{-}mol.C\text{-}mol^{-1**}$ | 0.15 (ref[8]) |
| Product yield coefficient | $Y_{p/x}$ | $C\text{-}mol.C\text{-}mol^{-1}$ | 2.5 (ref[8]) (based on lactate as an example metabolite) |
| Diffusivity of bulk nutrients | $D_{nutrient}$ | $m^2.s^{-1}$ | $6.7\times10^{-10}$ (ref [10]) (based on glucose as an example nutrient) |
| Diffusivity of metabolites | $D_{metabolite}$ | $m^2.s^{-1}$ | $1\times10^{-9}$ (ref[11]) (based on lactate as an example metabolite) |
| Bulk nutrient concentration | $S_b$ | $mM.L^{-1}$ | 4 (ref[12]) (based on glucose) |
| Biomass molecular mass | | $g.\ C\text{-}mol^{-1}$ | 27.8 (ref[8]) |
| Bulk nutrient molecular mass | | $g.\ C\text{-}mol^{-1}$ | 30.3 (glucose) |
| Metabolite molecular mass | | $g.\ C\text{-}mol^{-1}$ | 30.0 (lactate) |
| Bacterial diameter | | μm | 1 |
| Bacterial mass density | | $Kg.m^{-3}$ | 1100 (ref[13]) |
| Bacterial translational speed | | $\mu m.s^{-1}$ | 10 (refs [14,15]) |
| Bacterial rotational displacement | | degree | 45 |
| Duration of the planktonic phase | | s | 100 |
| Initial bacterial count (baseline simulation) | | | 200 |
| Simulation field size | | μm× μm | 500x500 |

| | | | |
|---|---|---|---|
| Simulation timestep interval | | s | 900 |
| Crypt height | | μm | 100 (refs[16,17]) |
| Crypt spacing (center-to-center) | | μm | 50 (ref[16], [17]) |
| Crypt diameter | | μm | 30 (ref[17,18]) |
| Oxygen minimal inhibitory concentration (Supplementary equation 1) | *MIC* | % | 6.61 (ref[1]) |
| Oxygen dependent growth rate – curve shape (Supplementary equation 1) | $\alpha$ | % | 0.764 (ref [1]) |
| Saturation constant for oxygen | $K_s$ (oxygen) | % | 0.12 (ref[1]) |
| Diffusivity of oxygen | $D_{O2}$ | $cm^2.s^{-1}$ | $1.53\times10^{-5}$ (ref [19]) |
| Number of invading bacteria | | | 100 |
| Duration of invasion | | s | 100 |

*Corresponding to baseline model
**Based on moles of carbon

# IV. SUPPLEMENTAL CODE DESCRIPTION

## ABM Code Implementation

### Simulation Overview

The agent-based model (ABM) represents bacteria as discrete spherical particles with variable mass, position, and species within a 2D box (500 μm×500 μm). For efficiency, simulation data is stored in the form of numerical arrays, with backend processing provided by the Numpy library.

The simulation is primarily made of a bacterial class storing all agent properties in NumPy arrays, in combination with classes representing global simulation states, such as physical environment, concentration gradients, and timestep. For implementation efficiency, the overall simulation is broken down into multiple modules that handle the physics within the system (concentration calculation, shoving and wall constraint, as well as replication and growth modules). After initialization, a time process module repeatedly steps the simulation forward by calling the physics modules in sequence and updating both agent and global states.

### Bacteria Agent Class

Upon initialization, bacteria are categorized by species with shared parameters like growth rate and density. Planktonic bacteria receive additional parameters governing speed, direction, and angular diffusion, which dictate their movement and rotation at each time step. All simulation parameters are detailed in Supplementary Table 1.

During runtime, each bacterium maintains individual values for position, biofilm status, species type, and mass. To enhance coding efficiency, each bacterium also has a Boolean 'existence' flag for quick deletion and a 'next_position' variable for easier collision handling.

### Field Class

The biofilm simulation is dependent on several continuously varying scalar fields. These include bacterial density, nutrient and metabolite concentrations and diffusion coefficients, and the presence or absence of rigid walls at a given location. For all such continuously varying fields,

simulation values are internally represented in the discretized form of 50x50 scalar matrices, with each element representing a (10 μm×10 μm) area within the simulation field.

**Concentration Solver Module**

The biofilm model allows for multiple chemical solutes to coexist in a steady state within the simulation, modeling bacterial metabolisms. Chemical concentrations are modeled as steady-state solutions to a partial differential equation in Equation 4.

Following the finite volume method, concentrations are approximated as constants within (10 μm×10 μm) grid blocks, forming a (50x50) lattice representation for biofilm chemical gradients. Each chemical field within a given simulation is then represented as a 50x50 numerical matrix, which may be solved using the off-the-shelf FiPy solver. We impose boundary conditions on all fields by constraining cell faces at the top of the volume to have a constant bulk concentration value, cell faces at the bottom to have zero flux, and side faces to have periodic boundary conditions. We also define diffusion coefficients for each cell within the simulation for each chemical species, with the specified values for nutrients and metabolites listed in our table of constants. Diffusion coefficients for substratum faces (including topographical features) are set to zero (except for materials metabolized by the host, for which diffusion is allowed through topographical faces at the normal rate to simulate emission). Bacterial concentrations within each cell are assumed to be constant for the purposes of solving for chemical concentrations within a single timestep. Bacterial concentrations for a given species are calculated within each finite volume block by summing the mass of each individual of such species within the block, normalizing by grid block volume, and storing the result in the corresponding entry of a discrete field.

Given chemical boundary conditions and bacterial concentrations, the PDE representing chemical concentrations within the biofilm system is completely represented by a joint differential equation for bacteria feeding and metabolic by-products (Equation 5). To extract chemical concentrations for a given timestep, we encode the metabolic PDE into FiPy's internal data structures by copying the field classes for diffusion coefficients and bacterial concentrations into FiPy mesh grids of the same dimensions. The full reaction-diffusion equation is entered in terms of DiffusionTerm and ImplicitSourceTerm components, and corresponds to the discretized version of Equation 5.

The FiPy solver approximates the joint differential equation as a linear system across the biofilm grid, which may introduce inaccuracies for highly non-linear behaviors such as bacterial feeding. As a result, a single solver iteration may not immediately converge upon an equilibrium solution for the biofilm PDE. By initializing the PDE with the solution from the previous biofilm timestep, the algorithm speeds up significantly (using the sweeping method). Solver iterations provide numerical values for the concentration level of each chemical species within the finite volume cells of our simulation, which are stored back into field arrays for further processing.

**Shoving Module**

The shoving module is a general-purpose solver for preventing significant bacterial collisions. It implements a shoving update algorithm for pushing bacteria apart when their volumes intersect. A shoving update roughly corresponds with physical pushing of bacteria, and needs to be iterated multiple times to fully eliminate overlaps.

During shoving, we iterate over each bacterium and identify every bacterium within a distance of twice the maximum possible bacterial radius as a potential neighbor. We then iterate over each bacterium and its neighbors and calculate the distance between them. Any bacteria with positions closer than the sum of their radii are considered intersecting and symmetrically shoved directly away from each other by a distance equal to one-half the length of overlap. Each shove is executed simultaneously, so bacteria overlapping with multiple others are pushed by the sum of each shoving vector. Positions resulting from shoving are accrued and stored as a potential next position for bacteria. If, at any point in shoving, a biofilm bacterium intersects with a planktonic bacterium, the planktonic bacterium is automatically converted to biofilm. The shoving method assumes a shoving factor of one based on previous models[4]. Multiple iterations of shoving (>10) result in the rapid elimination of overlaps.

**Wall Constraint Module**

Bacteria movements may be generated by planktonic motion or shoving. All potential movements are stored as a pair of positional coordinates, one for the current position and one for the candidate

next position. During movement, the wall constraint module may be applied to prevent bacteria from entering a solid wall.

At each movement step, a candidate next position is calculated based on planktonic motion or shoving. Then, the grid-block, where the bacterium will be located, is determined by taking the integer components of the $x$ and $y$ positions divided by the grid block size. Suppose the structural grid has a non-zero value at the grid-block indices. In that case, the bacterium is considered to be in collision with a wall, and the exact point along the line from the initial to the next position at which a collision would occur is calculated. Finally, the position value of the bacteria is set to this intersection point.

**Initialization**

Before simulation timesteps begin, bacteria are initialized in planktonic form, uniformly and randomly within the fluid environment. Initial numbers are a free parameter defined for each species, defaulting to two hundred for all simulations. On initialization, planktonic bacteria are uniformly and randomly assigned an angular direction. Bacterial movement at the side faces is wrapped around to the opposite side, while the upper face is reflective (assuming that the probability of bacteria exiting and entering the field is equal).

Finite volume cells for the simulation are initialized with a zero concentration for each solute. The top surface of the simulation field is constrained to the predetermined bulk concentration of each solute, while solid boundaries are assigned no-flux boundary conditions. When present, structural pillars are generated with a height of eight blocks (80 μm), thickness of two blocks (20 μm), and spacing of three blocks (30 μm).

**Timestep Module**

The timestep module sequentially calls physics and agent modules to simulate a single timestep of biofilm growth, representing roughly 15 minutes of elapsed time. A single timestep represents several joint updates to bacteria and environment, and can be grouped into three broad phases.

1. Planktonic Movement

   If, within the seeding phase, planktonic (non-biofilm) bacteria are introduced into the simulation at their default concentration. Their position is uniformly and randomly chosen from the area above the substratum.

   Planktonic bacterial positions are incremented in the direction of their orientation. To simulate angular diffusion, independent normally distributed variables with a mean of zero and a standard deviation of the species' angular diffusion rate are then added to each bacterial angular position. To prevent possible unphysical behavior, such as wall intersections, the wall constraint module is executed to cutoff movements at the point at which they intersect with a wall. Bacteria in contact with the wall are marked as biofilm participants and removed from the list of actively moving planktonic bacteria.

2. Physical Constraints

   Iteratively update bacteria position to prevent overlap between bacteria, or intersection with solid walls in the environment. A single iteration of shoving may not push all bacteria out of intersection with each other, so we repeatedly iterate shoving and wall constraint updates to fully push bacteria apart while preventing intersection with wall blocks. We find that nine iterations are consistently enough to resolve shoving or wall intersection conflicts during biofilm growth.

3. Concentration Solving

   Bacteria density is calculated within all grid block volumes for each species and is assumed to be constant for the purposes of concentration calculations within the timestep. The concentration solver module is applied to the joint equation for metabolic activity to solve for concentrations of each chemical.

4. Growth and Division

For each bacterial species within a given finite-volume grid block, the total growth rate may be calculated using the Monod equation as a function of chemical substrate concentrations (Supplementary Equation 3). We add an additional constant maintenance cost per unit mass to account for metabolic costs, giving the following equation for growth rate $\mu_{(k)}$ for bacterial species k in terms of chemical substrate concentrations $S_i$, maximum specific growth rate $\mu_{\max(k)}$ and equilibrium constants $K_{S(i,\,k)}$.

$$\mu_{(k)} = \left(\Sigma_i^l \mu_{\max(k)} \frac{S_i}{S_i + K_{s(i,\,k)}}\right) - m_k \text{ (Equation 3)}$$

where bacterium k uptakes substrates $S_i$ (i=1,2,…, $l$). All bacteria of species $k$ within the same finite volume then have their mass multiplied by a factor of $1 + \mu_{(k)}\Delta t$ to simulate the integration of mass growth along the simulation timestep interval $\Delta t$ . We assumed no upper bound for the growth rate, which may result in inaccuracies at high nutrient concentrations[5].

Bacterial radii are updated to have areas matching the new mass values, and bacteria with a radius greater than 2 μm are selected for splitting. Their mass is divided by two, and additional bacteria with the same mass and species are added at the same positions. The newly generated bacteria are then moved by the length of their diameter in a random direction from the location of the original. Finally, one last iteration of the wall constraint module is used to prevent newly generated bacteria from moving directly into solid surfaces.

## V. SUPPLEMENTARY REFERENCES


(1) Couvert, O.; Divanac'h, M.-L.; Lochardet, A.; Thuault, D.; Huchet, V. Modelling the Effect of Oxygen Concentration on Bacterial Growth Rates. *Food Microbiol* **2019**, *77*, 21–25. https://doi.org/10.1016/j.fm.2018.08.005.

(2) Zeitouni, N. E.; Chotikatum, S.; von Köckritz-Blickwede, M.; Naim, H. Y. The Impact of Hypoxia on Intestinal Epithelial Cell Functions: Consequences for Invasion by Bacterial Pathogens. *Molecular and Cellular Pediatrics* **2016**, *3* (1), 14. https://doi.org/10.1186/s40348-016-0041-y.

(3) Singhal, R.; Shah, Y. M. Oxygen Battle in the Gut: Hypoxia and Hypoxia-Inducible Factors in Metabolic and Inflammatory Responses in the Intestine. *J Biol Chem* **2020**, *295* (30), 10493–10505. https://doi.org/10.1074/jbc.REV120.011188.

(4) Lardon, L. A.; Merkey, B. V.; Martins, S.; Dötsch, A.; Picioreanu, C.; Kreft, J. U.; Smets, B. F. iDynoMiCS: Next-Generation Individual-Based Modelling of Biofilms. *Environmental Microbiology* **2011**, *13* (9), 2416–2434. https://doi.org/10.1111/j.1462-2920.2011.02414.x.

(5) Kovárová-Kovar, K.; Egli, T. Growth Kinetics of Suspended Microbial Cells: From Single-Substrate-Controlled Growth to Mixed-Substrate Kinetics. *Microbiology and Molecular Biology Reviews* **1998**, *62* (3), 646–666. https://doi.org/10.1128/mmbr.62.3.646-666.1998.

(6) Mitri, S.; Xavier, J. B.; Foster, K. R. Social Evolution in Multispecies Biofilms. *Proceedings of the National Academy of Sciences* **2011**, *108* (supplement_2), 10839–10846. https://doi.org/10.1073/pnas.1100292108.

(7) Estrela, S.; Brown, S. P. Metabolic and Demographic Feedbacks Shape the Emergent Spatial Structure and Function of Microbial Communities. *PLOS Computational Biology* **2013**, *9* (12), e1003398. https://doi.org/10.1371/journal.pcbi.1003398.

(8) Amaretti, A.; Bernardi, T.; Tamburini, E.; Zanoni, S.; Lomma, M.; Matteuzzi, D.; Rossi, M. Kinetics and Metabolism of Bifidobacterium Adolescentis MB 239 Growing on Glucose, Galactose, Lactose, and Galactooligosaccharides. *Applied and Environmental Microbiology* **2007**, *73* (11), 3637–3644. https://doi.org/10.1128/AEM.02914-06.

(9) Van Bodegom, P. Microbial Maintenance: A Critical Review on Its Quantification. *Microbial Ecology* **2007**, *53* (4), 513–523. https://doi.org/10.1007/s00248-006-9049-5.

(10) Kreft, M.; Lukšič, M.; Zorec, T. M.; Prebil, M.; Zorec, R. Diffusion of D-Glucose Measured in the Cytosol of a Single Astrocyte. *Cell Mol Life Sci* **2013**, *70* (8), 1483–1492. https://doi.org/10.1007/s00018-012-1219-7.

(11) Ribeiro, A. C. F.; Lobo, V. M. M.; Leaist, D. G.; Natividade, J. J. S.; Veríssimo, L. P.; Barros, M. C. F.; Cabral, A. M. T. D. P. V. Binary Diffusion Coefficients for Aqueous Solutions of Lactic Acid. *J Solution Chem* **2005**, *34* (9), 1009–1016. https://doi.org/10.1007/s10953-005-6987-3.

(12) Henson, M. A.; Phalak, P. Byproduct Cross Feeding and Community Stability in an in Silico Biofilm Model of the Gut Microbiome. *Processes* **2017**, *5* (1). https://doi.org/10.3390/pr5010013.

(13) Lewis, C. L.; Craig, C. C.; Senecal, A. G. Mass and Density Measurements of Live and Dead Gram-Negative and Gram-Positive Bacterial Populations. *Appl Environ Microbiol* **2014**, *80* (12), 3622–3631. https://doi.org/10.1128/AEM.00117-14.

(14) Saragosti, J.; Silberzan, P.; Buguin, A. Modeling E. Coli Tumbles by Rotational Diffusion. Implications for Chemotaxis. *PLoS ONE* **2012**, *7* (4), e35412. https://doi.org/10.1371/journal.pone.0035412.

(15) Cheong, F. C.; Wong, C. C.; Gao, Y.; Nai, M. H.; Cui, Y.; Park, S.; Kenney, L. J.; Lim, C. T. Rapid, High-Throughput Tracking of Bacterial Motility in 3D via Phase-Contrast Holographic Video Microscopy. *Biophys J* **2015**, *108* (5), 1248–1256. https://doi.org/10.1016/j.bpj.2015.01.018.
(16) Qi, X.; Pan, Y.; Hu, Z.; Kang, W.; M.d, J. E. W.; M.d, K. O.; Jr, M. V. S.; Rollins, A. M. Automated Quantification of Colonic Crypt Morphology Using Integrated Microscopy and Optical Coherence Tomography. *JBO* **2008**, *13* (5), 054055. https://doi.org/10.1117/1.2993323.
(17) Rudolph, S. E.; Longo, B. N.; Tse, M. W.; Houchin, M. R.; Shokoufandeh, M. M.; Chen, Y.; Kaplan, D. L. Crypt-Villus Scaffold Architecture for Bioengineering Functional Human Intestinal Epithelium. *ACS Biomater Sci Eng* **2022**, *8* (11), 4942–4955. https://doi.org/10.1021/acsbiomaterials.2c00851.
(18) Sumigray, K. D.; Terwilliger, M.; Lechler, T. Morphogenesis and Compartmentalization of the Intestinal Crypt. *Developmental Cell* **2018**, *45* (2), 183-197.e5. https://doi.org/10.1016/j.devcel.2018.03.024.
(19) Stewart, P. S. Diffusion in Biofilms. *J Bacteriol* **2003**, *185* (5), 1485–1491. https://doi.org/10.1128/JB.185.5.1485-1491.2003.
